# Protective CD4^+^ Th1 cell-mediated immunity is reliant upon execution of effector function prior to the establishment of the pathogen niche

**DOI:** 10.1371/journal.ppat.1009944

**Published:** 2021-09-20

**Authors:** Leah S. Hohman, Zhirong Mou, Matheus B. Carneiro, Gabriel Ferland, Rachel M. Kratofil, Paul Kubes, Jude E. Uzonna, Nathan C. Peters

**Affiliations:** 1 Snyder Institute for Chronic Diseases; Department of Microbiology, Immunology and Infectious Diseases, Cumming School of Medicine, Calgary, Alberta, Canada; 2 Department of Comparative Biology and Experimental Medicine, Faculty of Veterinary Medicine; University of Calgary, Calgary, Alberta, Canada; 3 Department of Immunology, Rady Faculty of Health Sciences, Max Rady College of Medicine, University of Manitoba, Winnipeg, Manitoba, Canada; 4 Department of Physiology and Pharmacology, University of Calgary, Calgary, Alberta, Canada; U.S. Food and Drug Administration and Center for Biologics Evaluation and Research, UNITED STATES

## Abstract

Intracellular infection with the parasite *Leishmania major* features a state of concomitant immunity in which CD4^+^ T helper 1 (Th1) cell-mediated immunity against reinfection coincides with a chronic but sub-clinical primary infection. In this setting, the rapidity of the Th1 response at a secondary site of challenge in the skin represents the best correlate of parasite elimination and has been associated with a reversal in *Leishmania*-mediated modulation of monocytic host cells. Remarkably, the degree to which Th1 cells are absolutely reliant upon the time at which they interact with infected monocytes to mediate their protective effect has not been defined. In the present work, we report that CXCR3-dependent recruitment of Ly6C^+^ Th1 effector (Th1_EFF_) cells is indispensable for concomitant immunity and acute (<4 days post-infection) Th1_EFF_ cell-phagocyte interactions are critical to prevent the establishment of a permissive pathogen niche, as evidenced by altered recruitment, gene expression and functional capacity of innate and adaptive immune cells at the site of secondary challenge. Surprisingly, provision of Th1_EFF_ cells after establishment of the pathogen niche, even when Th1 cells were provided in large quantities, abrogated protection, Th1_EFF_ cell accumulation and IFN-γ production, and iNOS production by inflammatory monocytes. These findings indicate that protective Th1 immunity is critically dependent on activation of permissive phagocytic host cells by preactivated Th1_EFF_ cells at the time of infection.

## Author summary

Ongoing chronic infection with the intracellular parasite *Leishmania major* results in robust protective immunity at sites of secondary challenge. However, translation of our understanding of this protective response into an efficient vaccine has remained elusive. This is due, at least in part, to the fact that primary chronic infection maintains multiple effector and memory subsets of lymphocytes, making it difficult to determine the relative importance of any single subset and their functional properties, including the time at which they mediate their effector function. Employing parabiosis, adoptive transfer, and *in-vivo* blockade of effector functions followed by analysis by intravital microscopy and flow cytometry, we find that the acute availability of circulating CD4^+^ T helper 1 effector cells (Th1_EFF_) at the time of secondary challenge is critical for the Th1 immune response to prevent *L*. *major*-mediated immunomodulation of host phagocytes and mediate protective immunity. Therefore, the present lack of effective Th1-mediated vaccines that target CD4^+^ memory T cell generation may be because protective Th1 immunity relies on preactivated Th1_EFF_ cells and IFN-γ availability prior to the establishment of a pathogen niche, which occurs before these memory T cells acquire effector function. To the best of our knowledge, our observations are the first to formally demonstrate the degree to which Th1_EFF_ cells are reliant on the time at which they interact with infected phagocytes to mediate protection and have significant implications for vaccine strategies against phagosomal infections such as *Leishmania spp*., *Mycobacterium tuberculosis*, *Salmonella enterica*, and *Crytococcus spp*..

## Introduction

CD4^+^ T helper 1 (Th1) cells are critical for protective immunity against the vector transmitted intracellular parasite *Leishmania major*, which targets dermal phagocytes as host cells for infection and replication resulting in cutaneous leishmaniasis. Despite this knowledge, conventional vaccination strategies targeting anti-*Leishmania* Th1 immunity have been largely unsuccessful and concomitant immunity, in which a chronic subclinical primary infection mediates a rapid Th1 response at a distal site of secondary challenge remains the gold standard of protective immunity in both humans and animal models [1–6]. Concomitant immunity has also been shown to play a role in other phagosomal infections such as *Mycobacterium tuberculosis*, *Salmonella enterica*, and *Crytococcus spp*., as well as chronic parasitic infections such as Malaria and helminthic infections, and cancer [7–16]. In the case of *Leishmania*, this protective concomitant response is characterized by a rapid accumulation of circulating Ly6C^+^T-bet^+^ Th1 effector (Th1_EFF_) cells at the secondary challenge site. These Th1_EFF_ cells are not derived from memory cells induced to proliferate by secondary challenge, are short-lived in the absence of an ongoing primary infection, can be recruited to the skin in an antigen-independent manner, and are ≥80% IFN-γ single-producing cells *in-vivo* [17]. At secondary sites this concomitant response is also associated with the rapid activation of iNOS^+^CCR2^+^ monocytes that facilitate parasite elimination [18,19] as well as heterologous protection against visceral forms of the disease in the spleen and liver caused by *L*. *infantum* [20].

Both concomitant and vaccine-induced Th1 immunity mediate accelerated responses against *Leishmania* challenge compared to naïve individuals [1,2,21]. However, when directly compared, the most defining feature of the protective concomitant response versus sub-optimal or non-protective responses elicited by conventional antigen-adjuvant vaccination is the rapid accumulation of IFN-γ producing Ly6C^+^Th1_EFF_ cells at the secondary site of infected sand fly challenge, which can be observed within hours and before *Leishmania* begins to divide within phagolysosomes [1,2,17]. In contrast, vaccine-elicited Th1 immunity mediated by T central memory (T_CM_) cells is not observed until 7 days post-challenge. Similarly, comparison of Th1_EFF_ versus Th1 T_CM_ cells derived from chronically infected mice also demonstrated that the delayed recruitment of T_CM_-derived T_EFF_ cells following clonal expansion in the dLN is either not protective or associated with sub-optimal protection [17,22]. These observations suggest Th1 cells must interact with phagocytes at the time of, or shortly after, *Leishmania* infection in order to mediate efficient protective immunity. Remarkably, the degree to which anti-*Leishmania* Ly6C^+^ Th1_EFF_ cells, or Th1 cells in general, are reliant upon interacting with infected phagocytes prior to the initiation of intracellular pathogen replication in order to mediate their protective effect is largely unknown. Rather, models of Th1 mediated pathogen elimination typically involve a scenario in which the pathogen undergoes replication in the phagolysosome before T cell production of IFN-γ mediates phagocyte activation and pathogen elimination. However, more recent observations suggest that rapid recruitment of circulating Ly6C^+^ Th1_EFF_ cells [1,17] or a combination of preexisting Th1_EFF_ cells and T resident memory (T_RM_) cells [23], or even T_RM_ cells on their own [19], are critical to mediate rapid activation of otherwise permissive monocytic host cells prior to *Leishmania* proliferation [18,19]. Observations in which prior non-specific genetic reprogramming of phagocytic progenitors [24] provides improved protective immunity have also emphasized the potential importance of phagocyte activation prior to the establishment of the pathogen niche. While the need for such a rapid response is not overtly obvious given the relatively slow growing nature of phagosomal and parasitic pathogens [18], or even cancer, these conditions are associated with a wide variety of immunomodulatory strategies to efficiently establish hospitable niches within their hosts and may be refractory to subsequent immunity if given sufficient time [18,25–28].

In the present work, we investigated the degree to which a protective CD4^+^ Th1 cell population was reliant upon the time at which they interacted with infected phagocytes to mediate protection against *L*. *major*. This information is critical because a lack of sufficient emphasis on the importance of the timing of Th1_EFF_ function and IFN-γ may be a contributing factor to the failure of many CD4 T cell-mediated vaccination strategies focused solely on the generation of memory cells with a Th1 phenotype rather than the time at which they activate phagocytes [29,30]. We report that circulating Ly6C^+^CD4^+^ Th1_EFF_ cell availability at the time of secondary challenge is required to prevent pathogen niche establishment in host phagocytes and represents a critical mechanism of protective concomitant Th1 immunity. Because Th1_EFF_ cells require persisting antigen to be maintained, we suggest that our observations provide the strongest evidence to date that the vaccination strategy most likely to succeed again phagosomal infections is one that maintains circulating Th1_EFFs_, such as a live-attenuated vaccine [31].

## Results

### Protection at sites of *Leishmania* re-challenge is associated with adaptive CD4^+^ T-cell immunity

**Fig 1:**
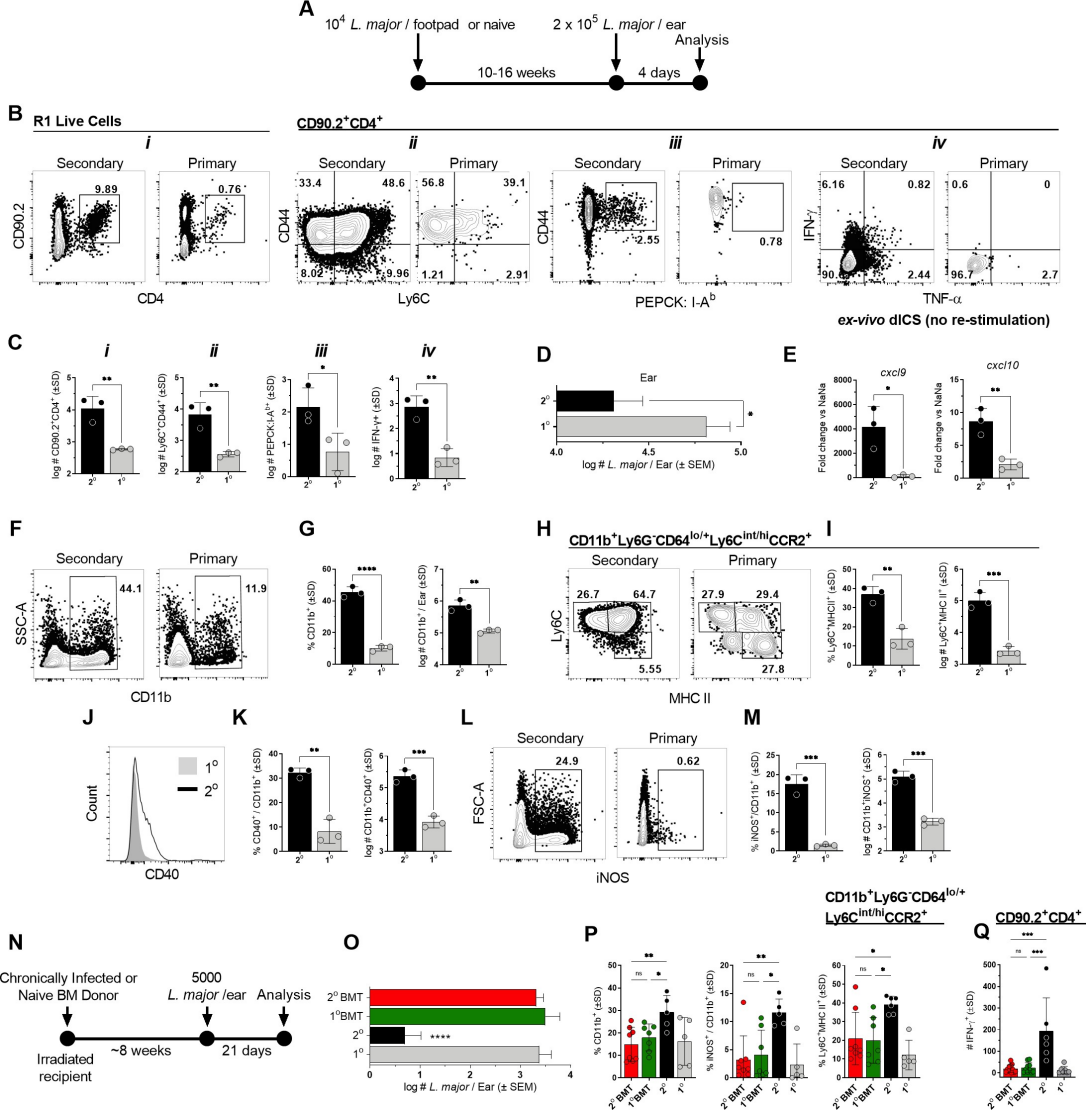
Protection at sites of *Leishmania* re-challenge is associated with adaptive CD4^+^ T-cell immunity. **(A-M)** Chronically infected (2^o^) or naïve (1^o^) age-matched controls (AMCs) were challenged via intra-dermal inoculation of 2 x 10^5^ *L*. *major* metacyclic promastigotes in both ears and the dermal site of challenge was analyzed at day 4 post-challenge (p.ch.) **(A)** Experimental design following the style of Butler et. al. (Cell Rep 2017). **(B)** Representative flow plots of the indicated dermal T cell populations. **(C)** Absolute number of the indicated T cell populations per ear. **(D)** Parasite loads in individual ears as determined by limiting dilution assay (LDAs). **(E)** Gene expression levels of *cxcl9* and *cxcl10* chemokines in bulk tissue by qRT-PCR. **(F, H, J, L)** Representative flow plots and **(G, I, K, M)** the frequency and absolute number of the indicated phagocyte populations per ear. **(N-P)** Naive congenic recipient mice were irradiated and transferred with 2 x 10^7^ bone marrow derived cells from chronic (2^o^ BMT) or naïve (1^o^ BMT) mice prior to challenge. **(N)** Experimental design. **(O)** Parasite loads in individual ears as determined by LDAs. **(P)** Frequencies of the indicated phagocyte populations. **(Q)** Absolute # of CD4^+^CD90.2^+^IFN-γ^+^ T cells per ear after overnight antigen re-stimulation. In **(A-M)** n = 3 mice/group/experiment, representative of 3 repeat experiments; in **(N-Q)** n = 5–8 total mice pooled from two independent experiments. (*) p<0.05, (**) p<0.01, (***) p<0.005, (****) p<0.0001, n.s. = not significant. Statistical analysis was performed using Student’s t-tests or one-way ANOVA with Tukey post-test.

In mice with a healed but chronic primary *L*. *major* infection at a localized site in the skin, optimal CD4^+^ T cell-mediated protective immunity at a distal site of dermal secondary challenge is associated with a rapid and robust increase in CD4^+^ T cell numbers at 4 days post-challenge (p.ch.) (Fig 1A–1C; [1,17,18]). Needle challenge elicits similar early IFN-γ-producing CD4^+^ T cell responses at the site of secondary challenge when compared to *Leishmania* infected sand fly challenge, the natural mode of infection (S1 Fig). Detection of CD4^+^ T cells with the capacity to produce IFN-γ at early time points following needle challenge also correlates with protection against insect vector mediated transmission of *Leishmania* [1,2]. Therefore, we analyzed immunity and parasite load within the first few days or weeks following needle challenge, an approach that we have argued is a valid pre-clinical model system to study protection [32].

A large number of the rapidly recruited CD4^+^ T cells at the secondary site of challenge express a CD44^+^Ly6C^+^ Th1 T_EFF_ cell phenotype [17], include *Leishmania*-specific CD44^+^PEPCK:I-A^b^ tetramer^+^ cells [33](S2 Fig) and cells producing IFN-γ *in-situ*, the detection of which does not require *ex-vivo L*.*m*. protein or pharmacological re-stimulation (Fig 1B and 1C; [17]. IFN-γ-mediated immunity results in a rapid ~5-fold reduction in parasite burdens by day 4 p.i. (Fig 1D), is associated with gene expression of the IFN-γ-inducible monocyte-recruiting chemokines *cxcl9* and to a lesser extent *cxcl10* (Fig 1E), and robust recruitment of CD11b^+^ phagocytic cells (Fig 1F and 1G), including increased frequencies and numbers of Ly6C^+^MHCII^+^ monocytes (Fig 1H and 1I; [18,19] (S3 Fig)). CD11b^+^ cells at secondary sites express higher levels of the activation marker CD40 (Fig 1J and 1K) and there is a 100X increase in the number of CD11b^+^iNOS^+^ cells (Fig 1L and 1M). While bone-marrow derived monocytes are essential for parasite control at re-challenge sites [18,19], protection in this model system was not mediated by permanent changes in phagocytic progenitors from mice with a healed primary infection, i.e., innate memory [24], as transfer of bone-marrow (BMT) from chronic mice conferred no protection in naïve, irradiated recipients, a methodology employed by us previously ([34], 2^o^ BMT group in Fig 1N and 1O), and resulted in no change in the frequencies of CD11b^+^, iNOS^+^CD11b^+^ or Ly6C^+^MHC II^+^ monocytes relative to intact naïve or naïve transfer controls (Fig 1P), all of which were significantly lower than intact chronic mice that have CD4^+^ T_EFF_, T_CM_ and T_RM_ cells [17,22,23]. Consistent with this, 2^o^ BMT recipients also showed no increase in the absolute number of IFN-γ^+^ CD4^+^ T cells (Fig 1Q) relative to naïve controls and had significantly lower numbers than intact chronically infected mice (2^o^), suggesting no impact on T cell priming.

### iNOS induction and protection is associated with CXCR3-mediated recruitment of CD4^+^ Th1_EFF_ cells

**Fig 2:**
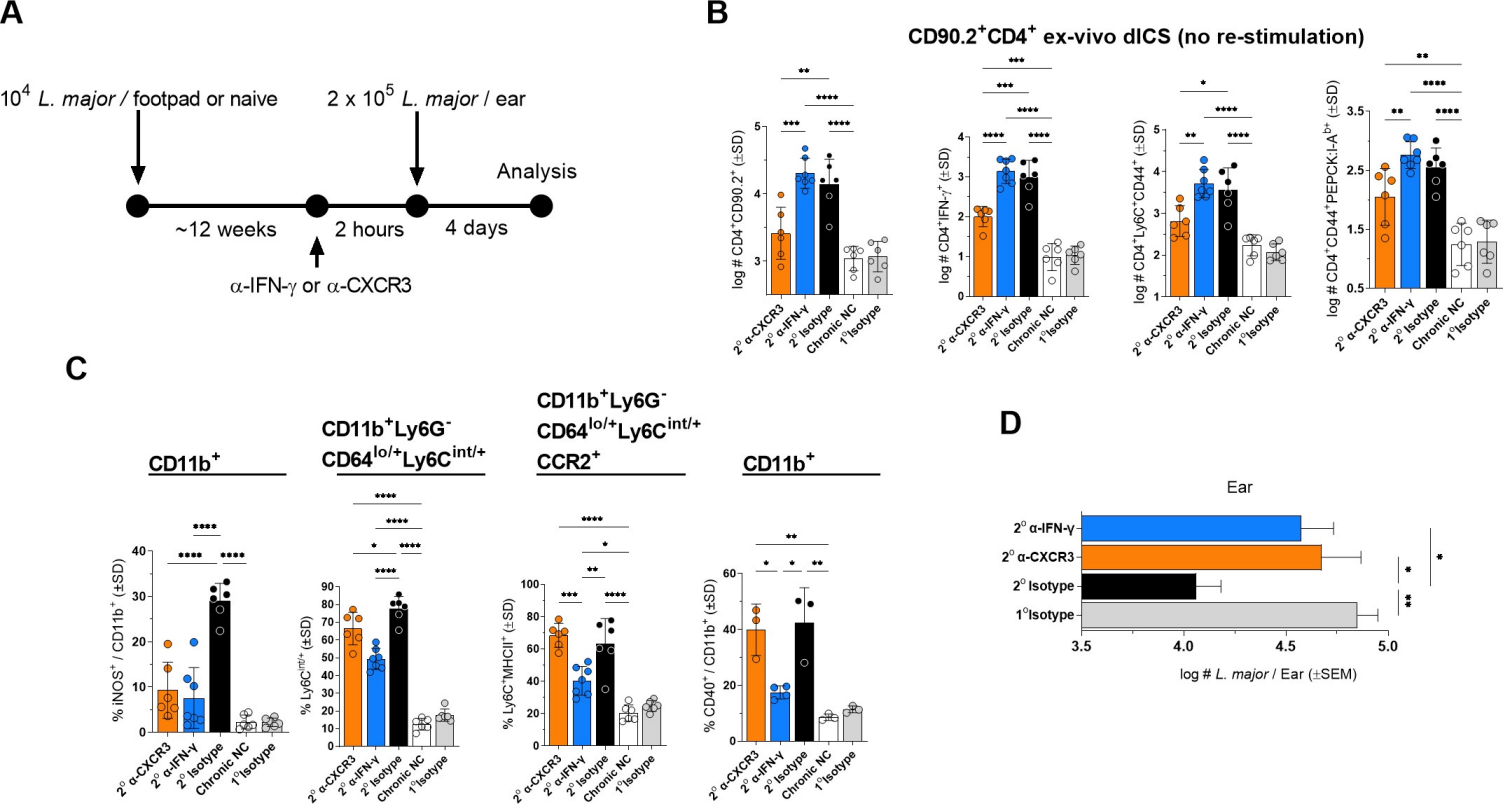
CXCR3-mediated recruitment of CD4^+^ T cells is critical for iNOS production and parasite control. **(A-D)** Chronic (2^o^) or naïve (1^o^) AMC mice were injected i.p. with 0.5mg anti-IFN-γ, 0.25mg anti-CXCR3, or 0.5mg HRPN control 2 hours prior to challenge as described in Fig 1A–1F. **(B)** Absolute # of the indicated CD90.2^+^CD4^+^ T cell populations per ear. **(C)** Frequencies of the indicated phagocyte populations. **(D)** Parasite loads in individual ears as determined by LDA. Each data point represents one mouse, n = 6–7 total mice pooled from two independent experiments except for the right panel in **(C)** %CD40^+^, n = 3–4 mice/group/experiment, representative of 2 repeat experiments. Statistical analysis was performed using one-way ANOVA with Sidaks post-test. Non-significant differences are not indicated.

Given the importance of T cell-derived IFN-γ in the control of phagosomal pathogens, we sought to assess the early drivers of IFN-γ production, iNOS induction, and parasite elimination. Both T_RM_ and circulating T_EFF_ cells, but not T_CMs_ or their progeny, have been identified as sources of early IFN-γ [17,23] and resident CD4^+^ T cells with the capacity to produce IFN-γ upon *ex-vivo* stimulation pre-exist at uninfected distal dermal sites in the chronically infected mice employed here (S4 Fig; [17]). Therefore, we assessed the impact of anti-IFN-γ (blocking both T_RM_ and circulating T_EFF_ IFN-γ-mediated effector function) versus anti-CXCR3 (blocking recruited T_EFF_ effector function only) mAb blockade on immunity at day 4 p.ch. (Fig 2A). As expected, anti-CXCR3 treatment successfully reduced total (5-fold), IFN-γ-producing (11.7-fold), CD44^+^Ly6C^+^ (6.6-fold), and PEPCK:I-A^b^ tetramer^+^ (2.6-fold) CD4^+^ T cells at the site of challenge (Fig 2B). In contrast, anti-IFN-γ treatment resulted in no difference in T cell recruitment relative to the isotype control, suggesting IFN-γ does not play a role in early T cell recruitment to a site of secondary challenge. In contrast, both anti-IFN-γ and anti-CXCR3 strongly reduced the frequency of iNOS^+^CD11b^+^ phagocytes to the same extent (Fig 2C, left panel), reduced the frequency of Ly6C^+^ monocytes, and abrogated protective immunity (Fig 2D), suggesting that IFN-γ derived from recruited cells is critical for early protection. In contrast, anti-IFN-γ, but not anti-CXCR3, reduced the frequency of activated Ly6C^+^MHCII^+^ monocytes and CD40^+^ CD11b phagocytes (Fig 2C), suggesting that IFN-γ derived from non-recruited tissue resident cells can alter the phenotype of recruited monocytes to a more mature Ly6C^+^MHCII^+^ phenotype, as previously shown [19], but this is not sufficient for early iNOS induction and parasite control under the conditions employed here.

### Circulating T_EFF_ cell effector function at the dermal site of re-challenge occurs independently of the tissue environment in chronic or naïve mice

**Fig 3:**
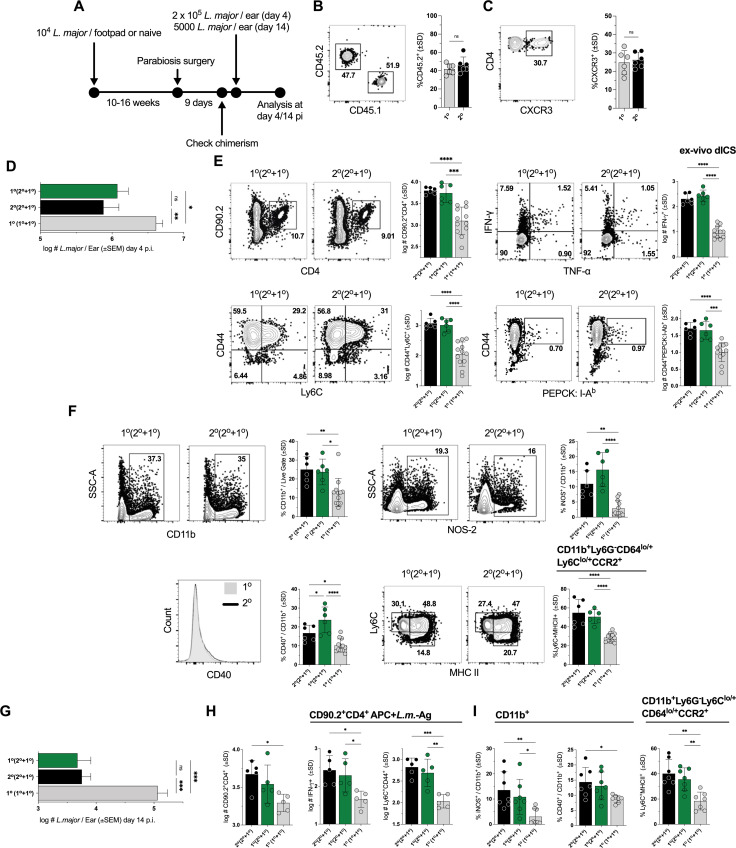
Circulating T_EFF_ cell effector function at the dermal site of re-challenge occurs independently of the tissue environment in chronic or naïve mice. **(A)** Chronically infected mice (2^o^) were surgically conjoined to naïve AMCs (1^o^) (2^o^ + 1^o^), or naïve AMCs were surgically conjoined with one another (1^o^ + 1^o^). **(B and C)** Frequency of 2^o^ mouse-derived CD45.2^+^ expressing CD45^+^CD4^+^ T cells **(B)** in samples in which CXCR3 expression **(C)** was also determined in both the 1° or 2° partner on day 9 post-surgery. Mice were then challenged with **(D-F)** 2 x 10^5^ or **(G-I)** 5000 *L*. *major* and analysis was conducted day 4 p.ch. **(D-F)** or day 14 p.ch. **(G-I). (D and G)** Parasite loads in individual ears as determined by LDAs. **(E and H)** Absolute number of the specified CD90.2^+^CD4^+^ populations per ear. **(F and I)** Frequency of CD11b^+^ phagocytic cells expressing the specified markers. Each data point represents one mouse; n = 6 (B and C) or 5–10 total mice/group pooled from 3 **(D-F)** or 2 **(G-I)** independent experiments. Statistical analysis was performed using one-way ANOVA with Tukey post-test.

We also utilized chronic-naïve (Ch-Na) parabiotic pairs to examine the potential impact of the tissue environment in chronically infected mice on the ability of circulating cells to mediate protection (Fig 3A). Under these conditions, parabiotic partners share circulating cells while T_RMs_ or other infection induced changes in the periphery are largely restricted to the chronic partner (S4 Fig). Prior to challenge we found equivalent frequencies of CD45.2^+^ cells derived from the chronic partner in the circulation of either the naïve or chronic mouse in Na-Ch pairs (Fig 3B), as well as equivalent levels of CXCR3 expression on CD45.2^+^ cells (Fig 3C), suggesting equivalent availability of chronic-partner-derived T_EFF_ cells in each partner mouse. At day 4 p.ch., both the naïve and chronic partner mice in Ch-Na pairs exhibited a significant reduction in parasite burden relative to naïve-naïve (Na-Na) paired controls and were not different from each other (Fig 3D). Ch-Na paired mice also showed no differences in absolute numbers of total CD4^+^CD90.2^+^, CD44^+^PEPCK-specific tetramer^+^, IFN-γ^+^, or CD44^+^Ly6C^+^ T cells, while showing significantly higher numbers relative to Na-Na controls (Fig 3E). In addition, no differences in the frequency of total CD11b^+^, iNOS^+^ or CD40^+^ total phagocytes, or Ly6C^+^MHCII^+^ monocytes were observed between the naïve and chronic partners whereas both were significantly higher than Na-Na controls (Fig 3F). To ensure that these observations were not restricted to early time points and the higher challenge doses employed for day 4 analysis, we also employed a low dose challenge (5000 *L*. *major*; [17]) and analyzed infection at day 14 p.i. (Fig 3A and 3G–3I). Consistent with our day 4 observations, both Ch and Na partners of Ch-Na pairs robustly reduced parasite burden relative to Na-Na controls and were not different from each other (Fig 3G). We also saw no difference in the number of CD44^+^Ly6C^+^CD4^+^ T cells or CD4^+^ T cells with the capacity to produce IFN-γ (Fig 3H). Lastly, the frequency of iNOS^+^ or CD40^+^ total phagocytes or Ly6C^+^MHCII^+^ monocytes in Ch versus Na partners of ChNa pairs was the same, while both retained elevated levels relative to NaNa paired controls (Fig 3I). These observations indicate that circulating Th1_EFF_ cells derived from the Ch partner mouse are critical mediators of protective immunity independently of the pre-existing chronic tissue environment, including T_RM_ cells, of the chronic partner.

### Acute availability of IFN-γ and circulating Th1_EFF_ cells is critical for *L*. *major* control

**Fig 4:**
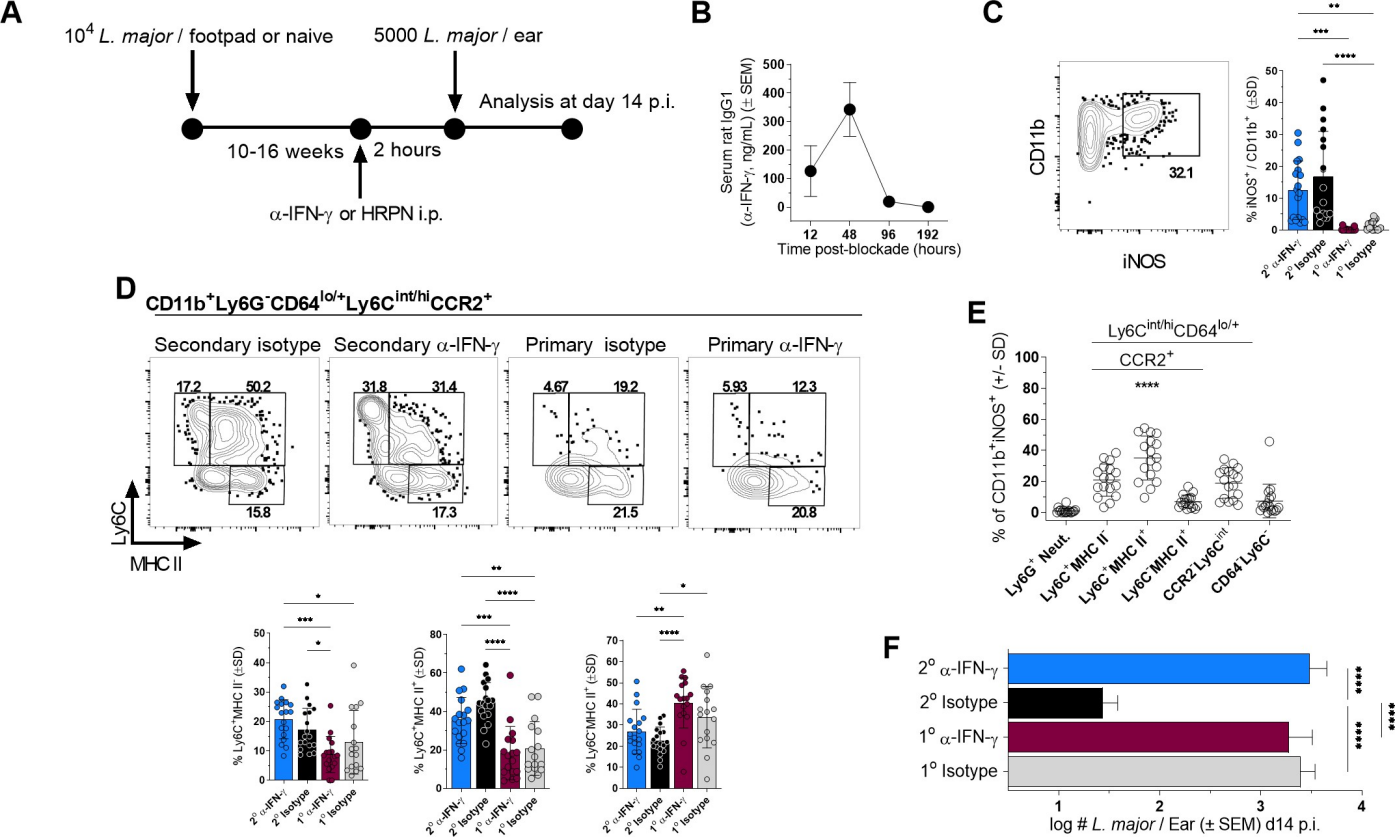
Early availability of IFN-γ is critical for *L*. *major* control. **(A-F)** Chronic mice (2^o^) or naïve (1^o^) AMCs were injected i.p. with 0.5mg anti-IFN-γ or HRPN control 2 hours prior to challenge with 5000 *L*. *major* and analysis was conducted on day 14 p.ch. **(B)** ELISA assessment of serum rat IgG1 (anti-IFN-γ ng/mL) from serum at the indicated time points post-anti-IFN-γ treatment. **(C and D)** Representative flow plots and frequency of CD11b^+^ cells expressing the specified phenotypic markers (D, lower panels) or iNOS (C, right panel) in the ear dermis. **(E)** Phenotype of iNOS^+^CD11b^+^ cells at the dermal site of *L*. *major* challenge in mice with a chronic primary infection. **(F)** Parasite loads as determined by LDA. n = 3 **(B)** or n = 11–18 **(C-F)**. Data is pooled from 3 independent experiments. Statistical analysis was performed using one-way ANOVA with Tukey post-test **(C, D, F)**. In **(E)** p<0.00001 versus all other groups.

Having defined circulating T_EFF_ cells as critical to optimal immunity, we next wished to address the relative importance of early IFN-γ production (≤4 days p.ch.), a period during which *L*.*m*. establishes infection but has not started to proliferate ([18]; S5 Fig). We first performed short-term transient IFN-γ blockade employing anti-IFN-γ treatment of chronic *L*.*m*. infected mice (Fig 4A; [18]) and assessed protection at d14 p.ch.. We employed a dose of blocking Ab that was cleared from the circulation by day 4 p.ch. (Fig 4B). Employing anti-CXCR3 in these experiments was not informative as the antibody was maintained in circulation for up to 14 days, well beyond the acute time frame of ≤4 days. Despite a recovery in the proportion of iNOS^+^CD11b^+^ cells (Fig 4C) and Ly6C^+^MHCII^+^ monocytes (Fig 4D), the predominant iNOS^+^ population (Fig 4E), early IFN-γ blockade resulted in a complete loss of protection at day 14 post challenge (Fig 4F). Therefore, despite the short-term presence of anti-IFN-γ and equivalent frequencies of iNOS^+^ phagocytes at the time of analysis, there was a clear, long term disruption in parasite control suggesting the early (≤d4) window is critical for protection mediated by IFN-γ producing CD4^+^ T cells.

**Fig 5:**
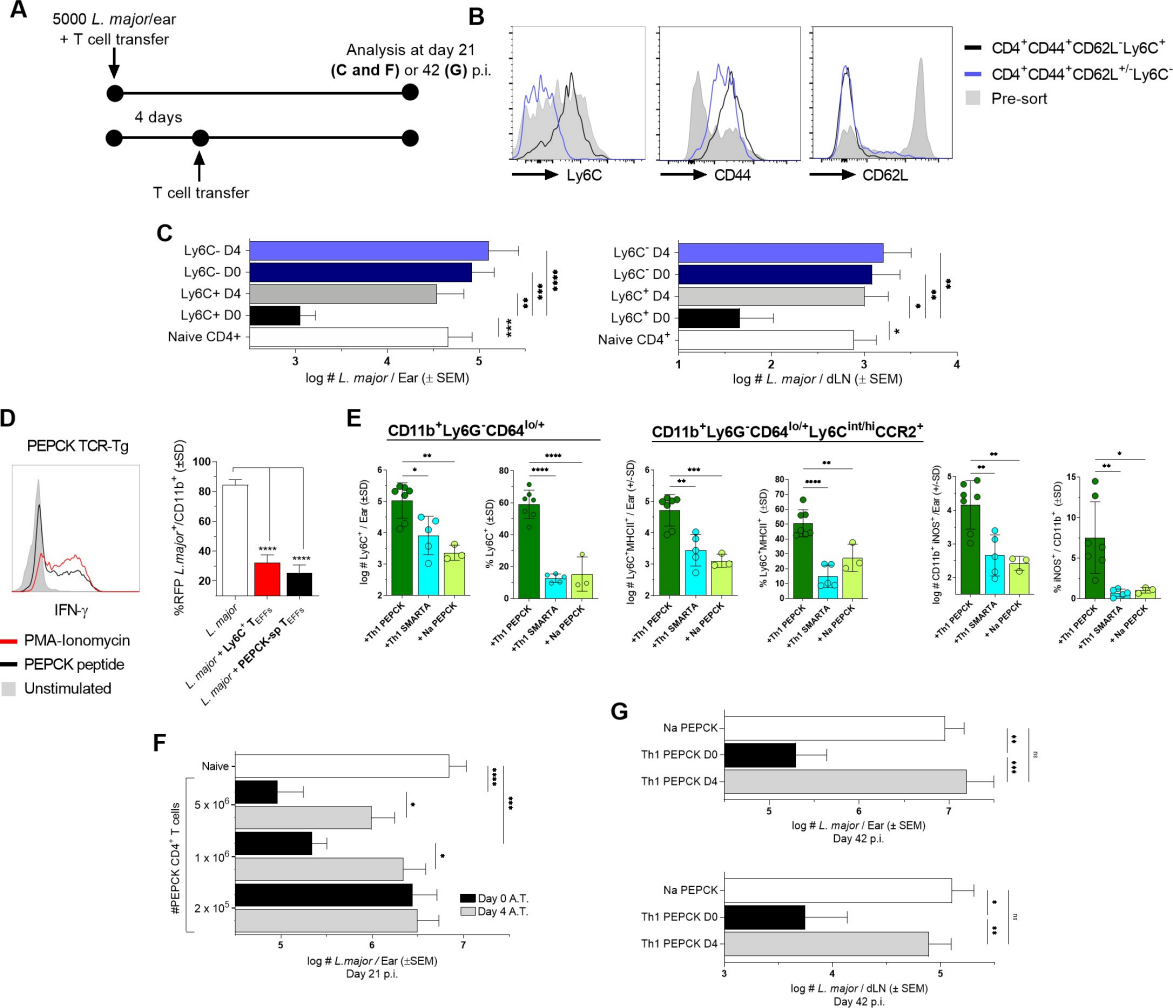
Circulating T_EFF_ availability prior to niche establishment is required for *L*. *major* control. **(A-G)** Naïve C57Bl/6 mice were infected with *L*. *major* and adoptively transferred immediately **(C, E-G)** or 4 days p.i. **(C, F, and G))** with **(C)** 2.5–3x10^6^ Ly6C^+^CD44^+^CD62L^-^CD4^+^ or 5.5–6x10^6^ Ly6C^-^CD44^+^CD4^+^ FACS sorted polyclonal T cells derived from the blood, spleen, and dLNs of chronically infected C57Bl/6 mice or **(E-G)** *in-vitro*-generated Th1 PEPCK-specific GFP^+^ TcR Tg T cells. **(B)** Analysis of Ly6C^+^, CD44^+^, and CD62L^-^ expression on polyclonal CD4^+^ T cells derived from chronically infected mice pre- and post-cell sorting. **(C)** Parasite loads in the ear dermis and ear dLNs following polyclonal CD4^+^ T cell transfer as determined by LDA at day 21 p.i.. **(D)** Representative histogram of IFN-γ^+^ Th1 PEPCK-specific *in-vitro*-generated T cells after PMA-Ionomycin, PEPCK peptide, or no stimulation (left panel) and comparison of %*L*. *major*-RFP *in-vitro* in bone marrow-derived monocytes by chronic mouse-derived Ly6C^+^CD44^+^CD62L^-^CD4^+^ T_EFFs_ or *in-vitro-*generated Th1 PEPCK-specific GFP^+^ TcR Tg T cells (right panel).. **(E)** Analysis of the frequency and absolute number of the indicated popuations on day 4 p.i. and adoptive transfer of 4-5x10^6^ Na or Th1 primed PEPCK TcR Tg or Th1 primed SMARTA TcR Tg CD4^+^ T cells. **(F)** Parasite loads in the ear dermis following Th1 PEPCK-specific transfer of the indicated number of cells as determined by LDA at day 21 p.i.. **(G)** Parasite loads in the ear dermis (top) and ear dLN (bottom) following transfer of 10^6^ Th1 PEPCK-specific cells at determined by LDA at day 42 p.i.. n = 3–11 **(D and E)** mice/group, 10–24 **(C and F)**, or 8–16 **(G)** total ears or dLN/group. Statistical analysis was performed using one-way ANOVA with Tukey **(D, E, and G)** or Sidek’s **(C and F)** post-test. Data is pooled from 3 **(C-F)** or 2 **(G)** independent experiments.

To further address the impact of timing on CD4^+^ T cell-mediated immunity, we adoptively transferred protective polyclonal CD44^+^Ly6C^+^CD4^+^ Th1_EFF_ cells derived from chronic mice into naïve congenic recipients via intra-venous (i.v.) inoculation immediately after or four days following infection and examined protection at day 21 p.ch. [17] (Fig 5A and 5B). CD44^+^CD4^+^ T cells depleted of the Ly6C-expressing Th1 T_EFF_ population were employed as a control. Polyclonal Ly6C^+^CD44^+^CD62L^-^CD4^+^ T_EFFs_ transferred on day 0 (approximately 30 minutes post-transfer) resulted in a robust reduction in parasite burden in both the ear dermis (site of challenge) and the ear dLNs at day 21 p.i. compared to mice transferred with CD4^+^ T cells from naïve mice (Fig 5C; [17]). In contrast, adoptive transfer of the Ly6C-depleted CD44^+^CD4^+^ T cell population, which contains T_CM_ and T effector memory (T_EM_) cells [17], provided no parasite control regardless of the time of transfer. Strikingly, transfer of Ly6C^+^ T_EFF_ at day 4 p.ch. completely abrogated their ability to reduce parasite numbers at day 21 p.ch., despite the fact that the parasite does not undergo significant expansion during this early 4 day window ([18]; S5 Fig), demonstrating that CD4^+^ T-cell mediated protection is highly dependent upon the early execution of effector function. Because this experimental design compares the functionality of the Ly6C^+^ Th1_EFF_ population against itself in settings of immediate (day 0 transfer) vs delayed (day 4 transfer) execution of effector function, our observations suggest that the difference in protective capacity is unlikely to be a product of T cell intrinsic antigen-specificity, cytokine producing potential, or homing capacity.

In order to investigate this further, we wished to determine whether an increase in the number of CD4^+^ T cells could overcome the requirement for the early execution of effector function, as increased quantities of cells is often a target of vaccination strategies. To more precisely control for antigen-specificity and protective capacity, we employed *Leishmania* glycosomal phosphoenolpyruvate carboxykinase (PEPCK_335-351_)-specific TcR Tg T cells [35] generated *in vitro* under Th1 polarizing conditions (Fig 5D, left panel), which were able to control *L*. *major*-RFP in infected monocytes equivalently to chronic-mouse derived Ly6C^+^ T_EFFs_ *in vitro* (Fig 5D, right panel). Adoptively transferred Th1 PEPCK T cells are able to mediate the correlates of protection observed in intact chronic mice when assessed at day 4 p.i. and transfer into naïve recipients, namely, increased numbers and frequencies of Ly6C^+^CCR2^+/-^ monocytes (46-fold increase), monocytes expressing the Ly6C^+^MHCII^+^ phenotype (40-fold), and iNOS^+^CD11b^+^ phagocytes (50-fold) versus Na PEPCK TcR Tg T cells and significantly greater than Th1-primed SMARTA TcR Tg T cells, the latter being specific for an unrelated viral antigen (Fig 5E). These observations confirm that *Leishmania*-specific Th1 T_EFF_ cells derived from the circulation can independently mediate monocyte recruitment and activation at the site of challenge. We then A.T.’d Th1 PEPCK T cells at varying doses immediately after or 4 days following *L*. *major* challenge of naïve recipients and analyzed parasite number on d21 p.ch.. When transferred at day 0, both 10^6^ and 5 x 10^6^ adoptively transferred PEPCK T cells were able to significantly reduce parasite burden relative to naïve *L*. *major* infected controls, while adoptive transfer of 2 x 10^5^ cells was insufficient to provide control (Fig 5F). These results indicate that a critical threshold of activated T cells is required at the time of infection to mediate protection. However, when transferred at day 4, PEPCK T cells were unable to reduce parasite burden even when large numbers of cells (5x10^6^, p = 0.071) were transferred, demonstrating that rapid effector function is critical for CD4^+^ T cell-mediated protective immunity and cannot be easily overcome with increasing doses of antigen-specific cells.

Lastly, to determine if PEPCK Th1 cells that underwent delayed transfer could recover if given more time to mediate protection we also analyzed parasite loads in recipient mice at 6 weeks post-challenge. We found that PEPCK Th1 cells transferred after the establishment of the pathogen niche once again failed to reduce parasite loads and harbored the same number of parasites as mice transferred with naïve PEPCK T cells (Fig 5G). These observations confirm that T_EFF_ cells are reliant on acute interactions with infected phagocytes to mediate their protective effect.

### Delayed availability abrogates the accumulation and activation of Th1_EFF_ cells at the dermal challenge site

**Fig 6:**
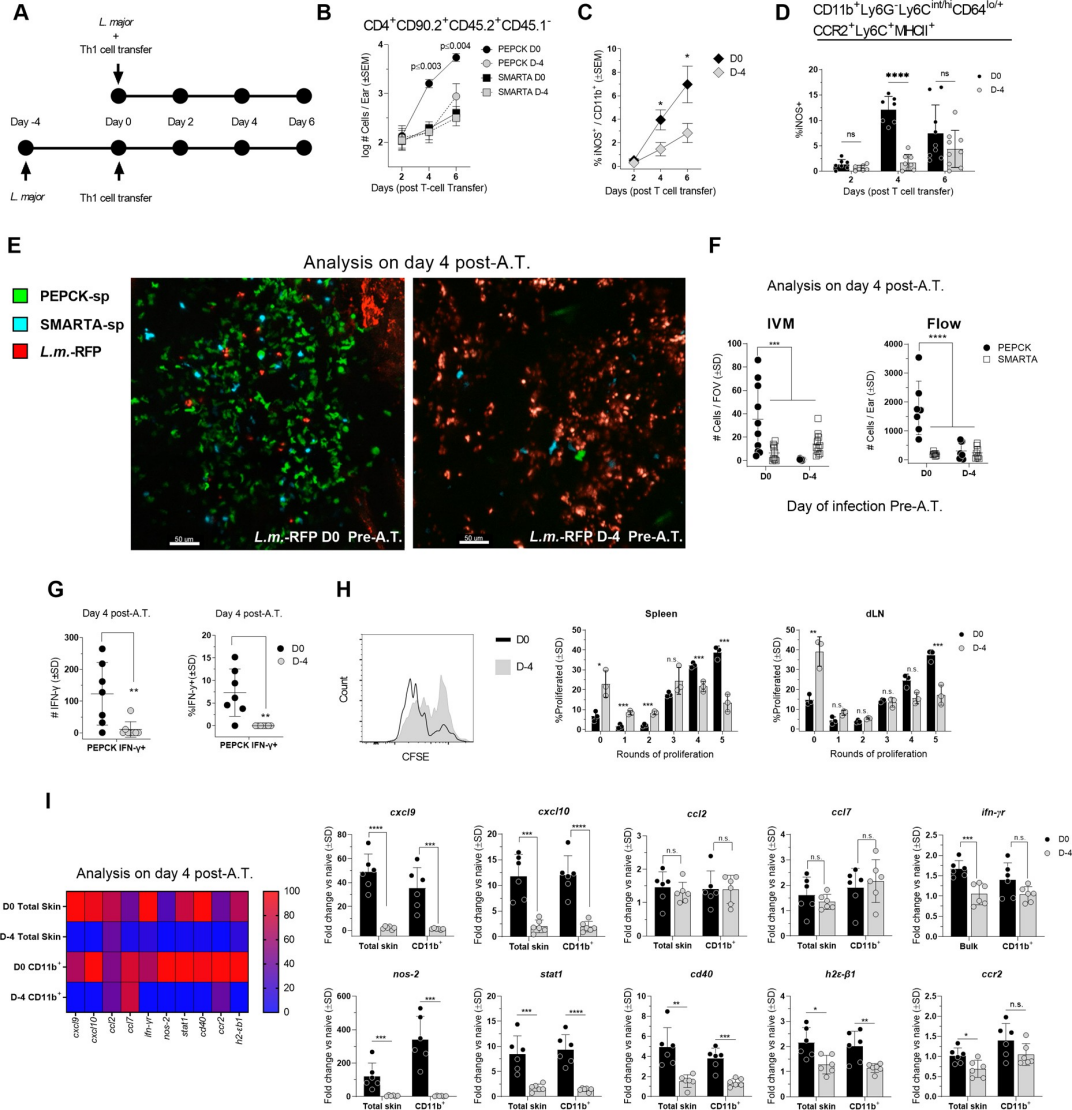
Delayed availability abrogates the accumulation and activation of Th1_EFF_ cells at the dermal site of challenge. **(A)** The indicated CD4^+^ Th1 cell populations were adoptively co-transferred into mice infected in the ear dermis with *L*. *major* just prior to (d0) or 4 days (D-4) prior to transfer and the immune response in the dermis was analyzed at an equivalent number of days post-transfer. **(B)** Absolute number of the specified T cell populations per ear on days 2, 4, and 6 post A.T.. 2-3x10^6^ GFP^+^PEPCK-specific Th1 T cells and 2-3x10^6^ SMARTA Th1 T cells **(C)** Frequency of iNOS^+^ cells within the total CD11b+ population. **(D)** Frequency of iNOS^+^ cells within the CD11b^+^Ly6G^-^Ly6C^int/hi^CD64^lo/+^CCR2^+^Ly6C^+^MHCII^+^ inflammatory monocyte population (see S6 Fig for other inflammatory monocyte populations). **(E)** Mice were challenged with *L*. *major* and adoptively co-transferred with 2.5-3x10^6^ CellTracker CFDA-labelled GFP^+^PEPCK-specific Th1 T cells and 2.5-3x10^6^ CellTracker Deep Red-labelled SMARTA Th1 T cells either immediately or at day 4 p.i.. Mouse ear dermis was imaged employing confocal intravital microscopy 4 days after T cell transfer.**(F)** Analysis of the number of T cells/field of view (FOV) (left panel) or the total number of T cells (right panel) 4 days after T cell transfer. **(G)** Analysis of the number of IFN-γ^+^ and frequency of IFN-γ^+^ Th1 PEPCK—specific CD4^+^ T cells at day 4 post-A.T. in the dermal site of challenge. **(H)** Mice were infected with 2 x 10^5^ *L*. *major* and adoptively transferred with ~3x10^6^ Na PEPCK-specific GFP^+^ T cells immediately or 4 days p.i.. Representative histograms of proliferation dye labelled PEPCK T cells and the % of cells within the indicated proliferation peak was analyzed on day 6 post-transfer in the spleen and ear dLN. **(I)** 4 x 10^6^ Th1 PEPCK-specific CD4^+^ T cells were transferred immediately or 4 days after mouse infection with 2 x 10^5^ *L*. *major*. Expression of the specified genes was assessed 4 days after T cell transfer with qRT-PCR and normalized against no-transfer *L*. *major* infected controls. n = 6–9 total mice/group pooled from 2 independent experiments except **(F, left panel)** in which n = 10 FOV were analyzed and data is representative of 2 independent experiments and **(H)** in which n = 3 and a single experiment representative of 3 experiments is shown. Statistical analysis was performed using Student’s t-tests, one-way ANOVA, or two-way ANOVA.

The loss of protective immunity observed when CD4^+^ T cell A.T. is delayed may be due to compromised T cell accumulation at the site of infection, or, if T cells are present, the ability of infected phagocytes to either elicit or respond to IFN-γ from these cells. To examine recruitment, we conducted a kinetic analysis of Th1-primed PEPCK versus SMARTA TcR Tg T cell accumulation in the skin at 2, 4, and 6 days following co-transfer into mice that were infected just prior to transfer (D0) or 4 days prior to transfer (D-4) (Fig 6A). SMARTA TcR Tg T cells were employed to act as an internal antigen-specificity control. At day 2 post T cell A.T., all T cells were found in equivalent numbers, regardless of the time of infection. However, PEPCK T cells transferred at the time of infection (D0) increased 14-fold between day 2 and 4 p-A.T. (p = 0.004), and this increase continued at day 6 (Fig 6B). In contrast, PEPCK T cells from mice infected on day -4 prior to A.T. did not increase in number between d2 and d4 post A.T., were present in significantly lower numbers on d4 and 6 post A.T., and were not significantly different from the number of Th1 SMARTA T cells. The inability of SMARTA cells to accumulate in the tissue strongly suggests that Th1 cells require cognate antigen recognition to accumulate in the tissue [36]. In the absence of Th1 PEPCK cell accumulation in the tissue, the frequency of iNOS producing CD11b^+^ cells was significantly reduced (Fig 6C), including iNOS production from Ly6C^+^ monocytes (Fig 6D). To extend these observations we focused on d4 post A.T. and employed intravital microscopy to visualize and enumerate cells *in-vivo* following co-transfer of Th1 primed PEPCK and SMARTA TcR Tg T cells. PEPCK T cells transferred at the time of challenge were visualized within the site of infection and in significantly higher numbers versus all other groups (Fig 6E and 6F, left panel). In addition, although significantly reduced in number (Mean 310 +/-SEM 105/Ear, n = 8), of the PEPCK^+^ cells that were present in the ear dermis of mice infected on day -4 prior to A.T. (Fig 6F, right panel), virtually none were producing IFN-γ *in situ* as assessed using our dICS assay that does not employ antigen or pharmacological restimulation (Fig 6G). The lack of IFN-γ production by this population is not due to the inability of these cells to make IFN-γ when sufficiently stimulated (Fig 5D and 5E). Rather, these data strongly suggest that, given time, those phagocytes that harbor the *Leishmania* parasite are compromised in their ability to act as effective antigen-presenting cells. In order to substantiate this observation, we A.T.’d naïve PEPCK TcR Tg T-cells into mice infected on day 0 or day -4 prior to transfer and determined the extent of T cell proliferation on day 6 post-transfer. Infections that were allowed to progress for 4 days prior to adoptive transfer were significantly compromised in their ability to drive PEPCK T cell proliferation in both the spleen and dLN as shown by reduced dilution of proliferation dye (Fig 6H). suggesting that established infections are compromised in their ability to prime naive *L*.*m*. antigen specific CD4 T cells.

To further assess the influence of parasite niche establishment on the immune environment we also examined gene expression 4 days after Th1 PEPCK T cell transfer (Fig 6I). Gene induction was normalized against control mice infected with *L*. *major* on day 0 or -4 to but did not receive A.T.. Mice with infections initiated on day -4 prior to transfer were significantly compromised in the expression of genes associated with protective Th1 immunity in preparations of both total skin and purified CD11b^+^ phagocytes, including *cxcl9*, *cxcl10*, *nos2*, *stat1*, *cd40*, *h2-εb1*, and to a lesser extent *ifnγr* and *ccr2*, indicating a significant abrogation of phagocyte effector function. Collectively, these observations suggest that acute Th1_EFF_ cell availability is required to capitalize on an early activation window in order to prevent the establishment of a pathogen niche.

### Infected monocytes are refractory to delayed of Th1_EFF_ effector function

**Fig 7:**
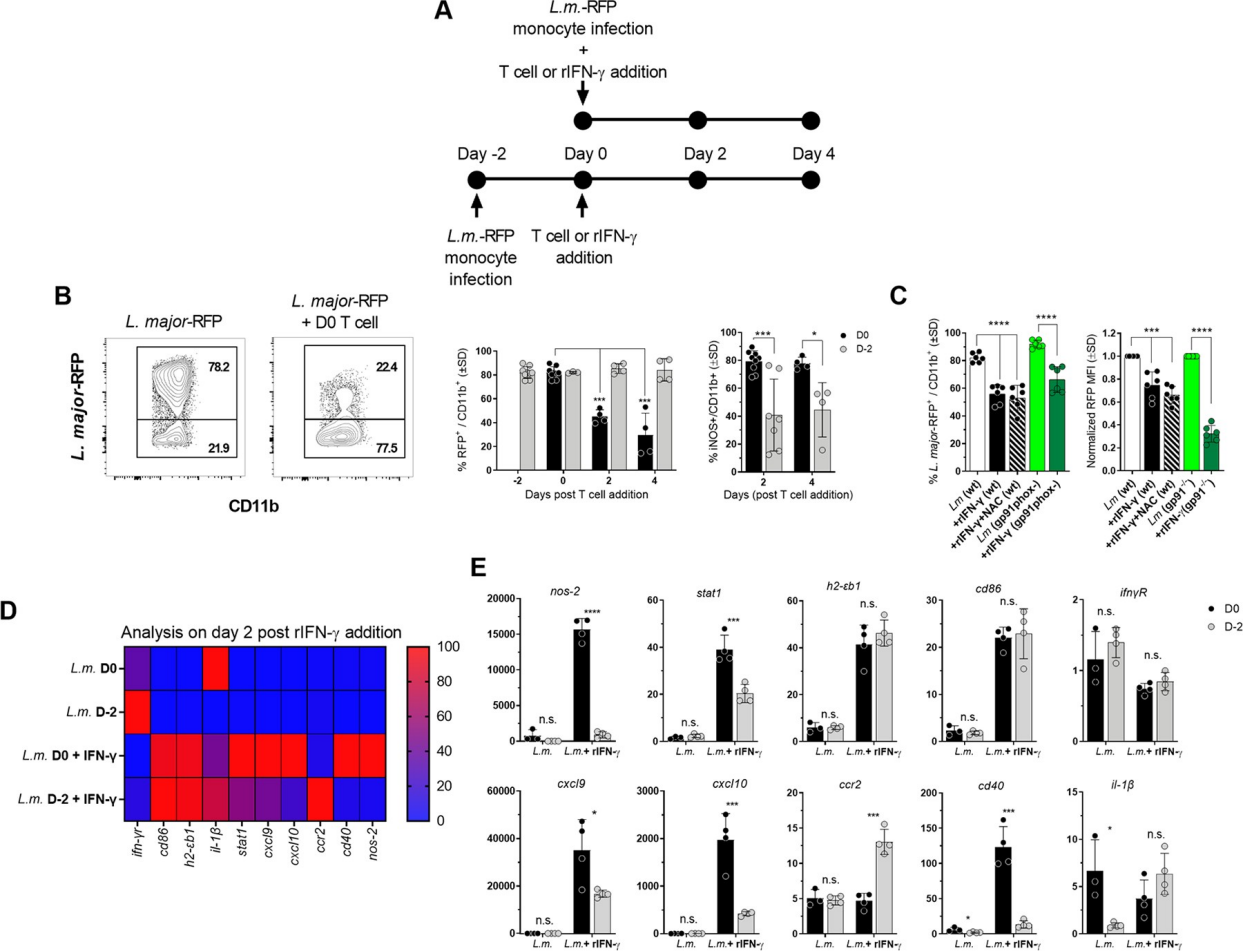
Infected monocytes are refractory to delayed of Th1_EFF_ effector function. **(A)** Purified bone marrow-derived primary monocytes were infected with *L*.*m*.-RFP, washed, and in-vitro-generated Th1 PEPCK-specific T cells were added immediately after or on day 2 p.i.. Monocytes were assessed at 2 or 4 days post-T cell addition. **(B)** Representative flow plots of CD11b vs *L*. *major*-RFP and frequency of RFP^+^ infected (left panel) and iNOS^+^ (right panel) monocytes at 2 or 4 days post-T cell addition. **(C)** Purified bone marrow-derived primary monocytes from wt or Phox^-/-^ mice were infected with *L*.*m*.-RFP. Indicated groups were treated with the ROS inhibitor NAC (N-acetyl-l-cysteine) at the time of infection. Recombinant IFN-γ was added immediately p.i.. Frequency of RFP^+^ infected monocytes and normalized RFP MFI in infected monocytes is shown. **(D)** Purified bone marrow-derived primary monocytes were infected with *L*.*m*.-RFP and recombinant IFN-γ was added immediately after or on day 2 p.i.. Monocytes gene expression was assessed via qRT-PCR 2 days after IFN-γ addition. Gene expression is shown via heatmap. **(E)** Individual gene expression between treatment groups. In **(B)** n = 4–9 representing two pooled experiments. In **(C)** n = 6 representing two pooled experiments. **(D and E)** n = 4 mice/group/experiment. Data is representative experiment of two repeat experiments.

To specifically demonstrate that, given time, the *Leishmania* parasite establishes a parasitic niche that down-regulates the ability of phagocyte-T cell interactions to control infection independently of T cell recruitment, we also employed *in-vitro* co-culture of PEPCK-specific Th1 T cells with *L*. *major*-RFP infected monocytes, the critical phagocytic cell involved in parasite clearance at secondary sites of infection (Fig 7A; [18,19]). Previous observations have demonstrated that prior exposure of macrophage cell lines to *Leishmania* altered their ability to subsequently respond to exogenous IFN-γ [37–39]. Because co-culture places infected phagocytes and T cells in the same physical space, this model system controls for the potential influence of trafficking in the *in-vivo* setting reported in Fig 6. Cultures were assessed at 2 and 4 days after T cell addition to monocytes which were infected just prior to (d0) or 2 days prior to co-culture (d-2) and parasite elimination was determined based on the frequency of RFP^+^ cells (Fig 7B). Th1 PEPCK T cell addition at day 0 resulted in a significant reduction in the frequency of RFP^+^ cells at day 2 and 4 post-co-culture, corresponding with a high frequency of iNOS^+^ monocytes (Fig 7B). In contrast, co-cultures initiated at day 2 post infection did not result in a reduction in the frequency of infected cells and significantly lower frequencies of iNOS^+^ cells, despite having the same amount of time to eliminate parasites, demonstrating Th1-primed *L*.*m*.-specific T cells are unable to eliminate parasites from infected cells over a 4 day period when monocyte-T cell co-cultures are initiated at day 2 p.i..

Given the observed requirement for rapid Th1 immunity, we hypothesized that the timing of iNOS expression may need to coincide closely with reactive oxygen species (ROS) oxidative burst to efficiently mediate parasite killing. To this end, we employed *gp91phox*^-/-^, wt, and wt + N-acetyl-l-cysteine (NAC), a free radical scavenger which inhibits ROS activity [40], pre-treated bone marrow derived monocytes and added r-IFN-γ immediately after *L*. *major*-RFP infection to assess differences in parasite control. Results were examined 2 days after r-IFN-γ addition and the %RFP^+^ and RFP MFI, to determine infection intensity on a per cell basis, of infected monocytes was assessed. No difference in %RFP^+^ cells or RFP MFI was observed between wt + rIFN-γ, wt + NAC + rIFN-γ, and *gp91phox*^-/-^ + rIFN-γ groups, suggesting that any potential role for ROS in immunity at a secondary site of challenge is not due to a requirement for close temporal proximity of ROS burst to IFN-γ-mediated iNOS induction (Fig 7C).

To further assess time dependent parasite-driven modulation of phagocyte responsiveness to IFN-γ, bone marrow derived monocytes were treated with r-IFN-γ following infection with *L*. *major* day 0 or day -2 of treatment. Addition of exogenous rIFN-γ in an *in-vitro* setting controls for the potential impact of both cell recruitment and the abrogation of infected phagocytes to act as efficient antigen presenting cells as reported in Fig 6G and 6H. Gene expression was assessed 2 days after r-IFN-γ addition (Fig 7D and 7E). Untreated cells did not have differences in expression levels of IFN-γ inducible genes. In contrast, addition of r-IFN-γ on day 0 p.i. induced significantly greater expression of the Th1 associated genes *stat1*, *cxcl9*, *cxcl10*, *cd40*, and *nos-2* relative to r-IFN-γ addition on day 2 p.i., while no differences in induction were seen for *ifn-γr*, *cd86*, and *h2b-εb1*. Of interest, expression of *ccr2*, which is associated with a less mature and more permissive monocyte phenotype was maintained at higher levels in monocytes infected at d-2 before r-IFN-γ addition. These results suggest that in instances of delayed IFN-γ availability, *Leishmania* establishes a pathogen-niche that abrogates the ability of infected monocytes to respond to IFN-γ.

## Discussion

Increased rapidity of the Th1 response at a site of secondary challenge with the phagosomal pathogen *Leishmania* is a strong correlate of optimal protective immunity and appears to be a prerequisite for protection against the more stringent conditions of physiological infected sand fly vector challenge [1,32]. This rapid response is predominantly mediated by pre-existing circulating Ly6C^+^ Th1_EFF_ cells [17]. Why this population is so protective is somewhat enigmatic, since T_EFF_ derived from clonally expanded T_CM_ cells in response to secondary challenge are functionally similar, although delayed in their response [17]. In this study, we isolated time as an experimental variable to demonstrate that protective CD4^+^ Th1 cells are critically dependent upon the time at which they mediate effector function. This was due, at least in part, to a requirement to execute Th1 effector function before establishment of a permissive pathogen niche in host phagocytes, specifically monocytes. We found that establishment of the pathogen niche within phagocytes negated their ability to elicit and mediate an otherwise protective Th1 immune response in multiple ways, including their ability to act as efficient APCs, upregulate Th1 associated genes, facilitate subsequent Th1 cell recruitment, and respond to IFN-γ.

It has been previously shown that high-dose anti-IFN-γ mAb treatment during early primary *Leishmania* infection results in a deficiency in the priming of Th1 immunity and enhanced disease [41,42]. Somewhat surprisingly, we also found that the highly protective response at a secondary site of challenge, mediated by already primed Th1 immunity, is also completely abrogated by early anti-IFN-γ treatment, despite an equivalent frequency of iNOS^+^ phagocytes at the time of parasite load analysis and well after (10 days) the clearance of the anti-IFN-γ XMG 1.2 Ab from circulation. A delay of Th1_EFF_ cell availability of only four days also completely abrogated their ability to control *Leishmania* expansion, thereby providing *in-vivo* evidence for a series of longstanding *in vitro* observations in which ‘M1-like’ classical activation of BM-derived macrophages by IFN-γ is compromised by prior infection [37–39,43]. The requirement for Th1 cells to be present at the time of infection suggests a scenario in which recently recruited and infected inflammatory cells that are not acutely exposed to IFN-γ provide a niche for the maintenance of infection and offers one possible explanation for why, even after the generation of robust Th1 immunity, sterilizing immunity against *Leishmania* is seldom achieved [44,45].

An important consideration when examining the possible sources of early IFN-y against phagosomal re- equivalent frequencies of CD45.2^+^ cells derived from the chronic partner in the circulation of either the e-existing Th1 effector and memory cells. Seeded T_RMs_ and circulating T_EFFs_ have been identified as early sources of IFN-γ whereas T_CM_, T_CM_-derived, or T_EM_ cells are not detected at early time points post-challenge and do not mediate the early control of parasite numbers associated with protection from infected sand fly challenge [17,22,23]. Our results demonstrate that the recruitment, induction of iNOS, and protection mediated by CXCR3-dependent circulating Th1_EFF_ cells occurred independently of the T_RM_-seeded chronic tissue environment, as previously suggested [17,22]. While iNOS production and parasite control was dependent upon both IFN-γ and CXCR3, emphasizing the critical role of recruited CXCR3^+^ Th1 T_EFF_ cells, anti-IFN-γ but not anti-CXCR3 resulted in reduced frequencies of MHCII^+^ monocytes, suggesting preexisting T_RMs_ can contribute to monocyte maturation in an IFN-γ dependent manner as reported by Glennie et. al. [19]. Why our observations employing anti-CXCR3 and parabiotic pairs showed a strong reliance on circulating T_EFF_ cells for early (4 days p.i.) control of parasites numbers versus the studies of Glennie et. al. [19], which implicated T_RMs_, may be related to the lower parasite doses employed in the current study or subtle differences in the manner in which the experiments were performed. In terms of the objective of the studies reported here, the use of circulating polyclonal Ly6C^+^ or *in-vitro* Th1-primed *L*.*m*. PEPCK-specific cells allowed for a tractable system to study the importance of the timing of Th1-monocyte interactions and it is important to note that our study did not directly assess the functionality of T_RMs_ in isolation_._ Therefore, we do not discount that these cells can be an early source of IFN-γ and may mediate a degree of parasite control when studied in isolation [19], albeit to a much smaller degree than that mediate by T_EFF_ cells.

The chemokine receptor CXCR3 plays a vital role in T cell recruitment and protective immunity against intracellular pathogens including *Leishmania* [46,47]. *M*. *tuberculosis* [48,49], *Salmonella enterica* [50], influenza [51]. and cutaneous leishmaniasis involves the production of multiple Th1-associated chemokines including CCL2, CCL7, CXCL9, and CXCL10 [18,23,52–57]. Given our observation that anti-CXCR3 as well as delayed A.T. abrogated protective immunity we hypothesized that Th1 cell accumulation at the site of challenge may be altered depending on the time at which T cells are initially available for recruitment. We found T cell recruitment at day 2 post-A.T. was equivalent regardless of the time of infection. This was similar to our observations in which early anti-IFN-γ blockade or comparison of naïve versus chronic challenge sites in naïve-chronic parabiotic pairs revealed no differences in acute T cell accumulation, suggesting that the initial influx of T cells into a site of challenge can be independent of both T_RM_ and IFN-γ. Previous observations demonstrating PBS can also drive the initial influx of circulating Th1 cells into a dermal challenge site [17], supports the conclusion that acute T_EFF_ recruitment is initially be driven by innate immunity in response to infection or injury, as recently suggested following *Aspergillus* infection [58]. In contrast, subsequent accumulation at sites of challenge on day 4 and 6 post-A.T. revealed significantly reduced PEPCK^+^ T cell numbers in mice infected on day -4 prior to transfer. Of significant interest, those *Leishmania*-specific T cells that did accumulate at the site of challenge following delayed A.T., albeit in 8-fold fewer numbers, were completely compromised in their ability to produce IFN-γ and adoptive transfer of naïve PEPCK T cells into mice with established infections significantly reduced their activation versus cells transferred at the time of infection. Therefore, the lack of accumulation of Th1-primed PEPCK^+^ T cells in mice with established infections may also be due to a lack of retention in the tissue in the absence of antigen recognition, as previously shown [36], in addition to the altered induction of subsequent T-cell mediated chemokine production, as demonstrated by the reduction in *cxcl9* and *cxcl10* gene expression at the day 4 timepoint in our A.T. model and shown in Fig 6I.

Monocyte recruitment and activation has been shown to be critical for elimination of *Leishmania* parasites at a dermal site of secondary challenge [18,19]. This is in contrast to neutrophils that harbor a significant proportion of those parasites that remain at the site of secondary challenge in settings of concomitant immunity, suggesting that even in settings of robust Th1 immunity neutrophils remain a safe haven for infection [18]. Here, we found that Th1 PEPCK T cells transferred at the time of infection mediated rapid upregulation of *cxcl9* and *cxcl10* gene expression, robust recruitment of protective Ly6C^+^MHCII^+^ and iNOS^+^ monocytes, and control of parasite numbers. Remarkably, transfer of otherwise protective T cells into mice infected on d-4 prior to transfer, or transient early blockade of IFN-γ in intact mice, significantly compromised the frequency iNOS^+^ monocytes and completely abrogated their protective capacity, even when these T cells were transferred in larger numbers. Therefore, in addition to the Th1-nature of protective CD4^+^ T cells, our observations formally demonstrate that the location and time at which they mediate their effector function is a critical component of their protective capacity and a formal correlate of protective immunity, on par with their Th1-phenotype and IFN-γ producing capacity. Therefore, future analysis of the induction of a protective immune state such as following prophylactic vaccination should extend beyond the mere presence of IFN-γ producing Ag-specific cells to include the time at which they mediate their effector function at the challenge site, including monocyte activation. We would argue that this is likely true for all pathogens that have converged upon the phagosome as a site of infection and replication.

Both iNOS and ROS represent major mechanisms of phagocyte control against phagosomal infections. To this end we hypothesized that the requirement for rapid immunity may be due in part to the relationship of these effector molecules. However, when the ability of infected wt, wt + NAC, and gp91^-/-^ monocytes to kill *L*. *major* in response to IFN-γ was examined, no differences were found in parasite control, suggesting that any potential role for ROS in immunity at a secondary site of challenge is not due to a requirement for close temporal proximity of ROS burst to IFN-γ-mediated iNOS induction. These findings are consistent with previous studies which have found ROS to play a negligible role in IFN-γ mediated parasite control during *Leishmania* spp. infection [59–62]

To date, naturally acquired concomitant immunity is the only response associated with protective immunity in humans against natural *Leishmania* infection via the bite of the infected sand fly vector [63–65]. These observations represent an important consideration for prophylactic vaccination against phagosomal pathogens which exhibit states of protective concomitant immunity, such as *Leishmania* [17,66], *M*. *tuberculosis* [67], and *S*. *enterica* [50], which are reliant on CD4^+^ Th1-mediated immunity and provide a possible explanation for why CD4^+^ T_CM_-targeting vaccination strategies have failed to replicate the degree of protection observed in concomitant immunity. The reliance of this response on an ongoing primary infection strongly suggests that the vaccination strategy most likely to succeed against phagosomal infections is one employing a design and route of inoculation that maintains a source of persisting antigen in the vaccinated host, such as a live attenuated vaccine, thereby maintaining a fast acting circulating Th1_EFF_ population [31,68].

## Methods

### Ethics statement

Ethics approval for this study was obtained from the Animal Care Committee (ACC) at the University of Calgary (Protocol number AC19-0007). Mice in this study were anesthetized using Ketamine or Ketamine-Xylazine prior to ear infection and/or parabiosis surgery, and received buprenorphine for analgesia following parabiosis surgery.

### Mice

C57BL/6 WT, B6.SJL-*Ptprc*^*a*^ *Pepc*^*b*^/BoyJ, B6.129S-*Cybb*^*tm1Din*^/J/gp91phox- and C57BL/6-Tg(UBC-GFP)30Scha/J mice were originally obtained from Jackson Laboratories. PEPCK.TCR-Tg mice [35] were kindly provided by Dr. Jude Uzonna (Department of Immunology, University of Manitoba, Winnipeg, MB). SMARTA.RAG^-/-^ mice were kindly provided by Dr. Markus Geuking (Snyder Institute for Chronic Disease, University of Calgary, Calgary, AB). PEPCK.TCR-tg(UBC-GFP) mice were bred in house. Mice were screened for TCR transgene expression using PCR. All mice were bred and maintained at the University of Calgary Animal Resource Centre under specific pathogen-free conditions.

### Parasites and parasite quantification

*Leishmania major* Friedlin (FV1) or *L*. *major*-RFP were maintained and metacyclic promastigote purification was performed as previously described [1,69]. Parasite loads were determined by limiting dilution analysis (LDA). Briefly, two-fold serial dilutions in 96-well flat bottom polystyrene microtiter plates were performed by administering 100μL of diluted tissue suspension to 100μL M199 complete medium. Plates were scored microscopically for growth and the number of parasites in each tissue was determined from the highest dilution well at which parasites were present after 7–10 days of incubation at 26°C.

### Primary infection and secondary challenge

Chronically infected mice were generated by injecting 10^4^ *L*. *major* metacyclic promastigotes s.c. in the left hind footpad in a volume of 40μL. Mice were either challenged when footpad lesions had completely resolved (10–16 wks p.i.) or employed as a source of polyclonal donor cells when lesion were beginning to resolve (6–8 weeks p.i.. Naïve mice and mice with a chronic primary infection were challenged with either 2x10^5^ or 5000 *L*. *major* metacyclic promastigotes i.d. in the ear in a volume of 10μL. In some experiments *L*. *major-*RFP was employed [70]. For imaging experiments, 20μL of *L*. *major*-RFP at 1x10^6^/ml was applied to the ventral ear surface and 20 i.d. ear pricks were conducted employing a sterile 20GA needle.

### Tissue processing

Ear, dLN, spleen, and blood were prepared as previously described [17,18,71]. Briefly for ears, ventral and dorsal sheets were separated, then submerged in 0.5-1mL DMEM containing 16μg/mL of Liberase and incubated at 37°C for 90–120 minutes. In experiments employing direct intracellular staining (dICs) of ears, ventral and dorsal sheets were place in 0.5-1mL pre-warmed DMEM containing 16μg/mL of Liberase and 20μg/mL Brefeldin A. Following Liberase treatment ear tissue was homogenized for 3.5 minutes in a Medicon using a Medimachine (Becton Dickson). Tissue homogenates were then flushed from the medicon with 8mL DMEM with 0.05% DNase I and filtered using a 50μm-pore-size cell strainer. In experiments employing dICs, 2μg/mL Brefeldin A was added to pre-warmed DMEM+DNase I media, and after flushing and filtering the ear homogenate was returned to 37°C for an additional 2 hours. Ear draining lymph nodes (dLNs) were removed and homogenized with a 1mL syringe plunger on a 70μm cell strainer. Spleens were removed and homogenized with a 1mL syringe plunger on a 70μm cell strainer. Red blood cells (RBCs) in the spleen were eliminated using ACK lysing buffer (Lonza). Blood was obtained by intra-cardiac bleed and T cells were isolated via Histopaque gradient isolation when used for adoptive transfer. For tail-nick bleeding, mice were warmed under heat lamps and then tails were dipped in warm water. Tail veins were nicked with scalpels and 3–10 drops of blood were collected. Serum was isolated from tail nick blood via centrifugation at 2000rpm for 10 minutes at room temperature and used for ELISA. To check for chimerism following parabiosis or bone-marrow chimeras, flow staining was performed directly on whole blood samples, then RBCs were removed using 10X RBC lysis buffer (eBioscience).

### CD4^+^ T cell purification and Th1 culture

GFP-PEPCK-specific and SMARTA CD4^+^ T cells were isolated from spleens using CD4 (L3T4) microbead magnetic bead separation (Miltenyi Biotec) according to product protocol. After CD4^+^ T cell isolation, samples were seeded in 96-well round bottom polypropylene plates at 10^5^ CD4^+^ T cells in 200μL of IMDM (10% FBS, 2mM L-glutamine, 1% Pen/Strep, 50μM 2-mercaptoethanol) with 2μL of Dynabeads Mouse T-Activator CD3/CD28 (Gibco) and 20ng/mL IL-12 (eBioscience) and 10U/mL IL-2 (Gibco). Cells were grown for 4 days at 37°C, with 100μL media removed at day 2 of culture and refreshed with 100μL of media containing fresh IL-2 and IL-12.

### *In Vivo* blocking antibody treatment

For short term inhibition of IFN-y and/or CXCR3 *in vivo*, mice were treated with a single intraperitoneal injection of 0.5mg *InVivo*MAb anti-mouse IFNy (XMG1.2) (BioXCell) or 0.25mg *InVivo*MAb anti-mouse CXCR3 (CXCR3-173) (BioXCell) 2 hours prior to *L*. *major* intradermal challenge in the ears. Control mice were treated with isotype controls in an identical manner. Anti-IFN-γ control mice were treated with 0.5mg *InVivo*MAb rat IgG1 isotype control, anti-horseradish peroxidase (HRPN) (BioXCell), anti-CXCR3 control mice were treated with 0.25mg *InVivo* MAb polyclonal Armenian hamster IgG (BioXCell).

### PEPCK_335-351_:I-A^b^ tetramer straining

PEPCK_335-351_:I-A^b^ tetramer straining and magnetic enrichment were performed as previously described [17,72]. Briefly, a single cell suspension was prepared from a single spleen, dLN, or from the ear and stained with phycoerythrin-labeled PEPCK_335-351_:I-A^b^-streptavidin tetramers for 1 hour at room temperature. Spleen and dLN samples were chilled to 4°C and incubated with magnetic anti-phycoerythrin beads for 30 minutes. Spleen and dLN samples were then washed with 5-10mL FACs buffer (PBS + 0.01% FBS) and run through a magnetized LS column (Miltenyi Biotec).

### Monocyte isolation, infection, and culture

Naïve C57Bl/6 femurs and tibias were removed and bone marrow was flushed using ~10mL of chilled PBS. Bone marrow was further homogenized with the plunger of a 1mL syringe and filtered through a 70μm-pore-size cell strainer. Monocytes were isolated from bone marrow using an EasySep Mouse Monocyte Isolation Kit (STEMCELL) according to product protocol. Monocytes were infected at a ratio of 1 monocyte:4 *L*. *major*-RFP metacyclic promastigotes for 6 hours at 37°C at 10^5^ monocytes/100μL RPMI (10% FBS, 2mM L-glutamine, 1% Pen/Strep, 1% Hepes). After infection, monocytes were washed with 5-10mL RPMI and spun at 1400rpm for 10 minutes at 4°C to remove extracellular parasites. Monocytes were then cultured with T cells at a 1 T cell:2 monocyte ratio for 2, 4, or 6 days at 10^5^ monocytes/100μL RPMI. Monocytes treated with R-IFN-γ were exposed at a concentration of 50U/mL after parasites were washed off (6 hours p.i.) or 2 days p.i. NAC treated monocytes were exposed to NAC at a concentration of 1000μM at the time of infection.

### Cell sorting and adoptive transfer

For adoptive T cell transfer, pooled single cell suspensions from the spleen, dLNs, and blood of chronic CD45.2^+^ C57Bl/6 were stained with a combination of CD4, CD62L, CD44, CD90.2, and Ly6C and purified using a FACsAriaII (BD Biosciences) cell sorter at the Nicole Perkins Microbial Communities Core Lab. Sorted populations were transferred independently by intravenous injection into CD45.1^+^ B6.SJL-*Ptprc*^*a*^ *Pepc*^*b*^/BoyJ congenic naïve recipient mice. The number of transferred cells is indicated in the text. Recipient mice were challenged either ~15 minutes or 4 days before adoptive transfer.

### Parabiosis

Parabiosis was modified from a previously described protocol [73]. Briefly, corresponding lateral sides of naïve or chronically *L*. *major* infected CD45.2^+^ C57Bl/6 mice and naïve CD45.1^+^ B6.SJL-*Ptprc*^*a*^ *Pepc*^*b*^/BoyJ mice were shaved. Matching skin incision were made at the thigh muscles of each mouse, and blunt dissection was performed up to the shoulder muscle to create ~0.5cm of free skin. Shoulder and thigh muscles were connected by continuous interrupted stiches, and the dorsal and ventral skins were connected by continuous stiches using chromic gut 5–0 (Ethicon). Mouse connection was further enforced via the application of external autoclips. Chronic(CD45.2^+^)-Naïve(CD45.1^+^) and Naïve(CD45.2^+^)-Naïve(CD45.1^+^) were weight-matched and had been previously co-housed for at least 2 weeks. Chimerism was checked after nine days based on CD45.1^+^ and CD45.2^+^ distribution between animals in tail nick blood samples. Once chimeric, mice were challenged with *L*. *major*.

### Chimeric mice

Bone marrow (BM) was harvested from chronically *L*. *major*-infected or naïve CD45.2^+^ C57Bl/6 mice. Femurs and tibias were removed and bone marrow was flushed using ~10mL of chilled PBS. Bone marrow was further homogenized with the plunger of a 1mL syringe and filtered through a 70μm-pore-size cell strainer. 2 x 10^7^ isolated BM cells were intravenously injected into CD45.1^+^ B6.SJL-*Ptprc*^*a*^ *Pepc*^*b*^/BoyJ recipients 2–4 hours post-irradiation with 9.5 Gy. Mice were kept under antibiotic treatment (2mg/mL Neomycin drinking water) for ~ 2 weeks post-irradiation and reconstitution. Chimerism was checked after ~8 weeks based on CD45.1^+^ and CD45.2^+^ distribution between animals in tail nick blood samples. Once chimerism was confirmed, mice were challenged with *L*. *major*.

### Re-stimulation of T cells for cytokine analysis, immunolabelling and flow cytometry

Single-cell suspensions were re-stimulated as previous described [17]. Briefly, single-cell suspensions were re-stimulated at 37°C in 5% CO_2_ for 14 total hours in flat-bottom polystyrene 48-well plates with 0.5–1 x 10^6^ T cell-depleted (STEMCELL) naïve spleen cells (APCs), with 50μg/mL freeze-thaw *Leishmania* antigen (*L*.*m*.-Ag). During the last 7 hours of culture, Brefeldin A was added to the wells at a final concentration of 1μg/mL.

For immunolabelling, cells were washed and labeled with fixable viability stain 510 (BD Biosciences) to exclude dead cells and anti-Fc III/II (CD16/32) receptor Ab (2.4G2) for 30 minutes at 4°C in the dark. In some experiments, cells were then stained with FITC anti-CX3CR1 (SAO11F11) and/or AF700 or BV421 anti-CCR2 (SA203611) for 30 minutes at 37°C in the dark. Following this, samples were stained with various combinations of the following antibodies at for 20 minutes at 4°C in the dark: PerCPCy5.5 anti-CD11b (M1/70); APC-Cy7 or PerCPCy5.5 anti-Ly6C (AL-21); FITC or AF700 anti-Ly6G (1A8); BV605 anti-MHC II (M5/114.15.2); BV786 anti-CD11c (HL3); APC, BV421, or BV786 anti-CD40 (3/23); BV650 or FITC anti-CD86 (GL1); PE-Cy7 or BV711 anti-CD64 (X54-5/7.1); AF647 anti-CD206 (MR5D3); PerCP-eF710 anti-KLRG1 (2F1); BV421 anti-PD-1 (29F.1A12); AF647 anti-CD121α (35F5); AF700, PerCPCy5.5, or PE-Cy7 anti-CD62L (MEL-14); FITC, PE-Cy7, BV786, or APC anti-CD90.2 (53–2.1 or 30-H12); AF700, APC, or PerCPCy5.5 anti- CD8 (53–6.7); BV605, PE-Cy7, APC, or BV421 anti-CD4 (RM4-5 or GK1.5); BV786, PE, APC-R700, or FITC anti-CD44 (IM7); APC or AF700 anti-CD45.2 (104); PerCPCy5.5 anti-CD45.1 (A20). In some experiments, cells were then fixed with BD Cytofix/Cytoperm (BD Biosciences) according to product protocol and stained with PE, APC, APC-Cy7, or BV786 anti-IFN-γ (XMG1.2) and BV650, APC, or FITC anti-TNF-α (MP6-XT22); or APC or PE-Cy7 anti-NOS-2 (CXNFT) for 45 minutes at 4°C in the dark. All Abs were from BD Biosciences, eBiosciences, Biolegend, or R&D systems. Data were collected using FACsDiva software on a FACSLSRII or FACSCANTO II flow cytometer (BD Biosciences), and analyzed using FlowJo software (TreeStar). Forward-scatter-area and forward-scatter height was employed to exclude cell doublets from analysis. To determine the absolute number of cells, a portion of each sample was removed for counting with 123count eBeads Counting Beads (Invitrogen) as previously described [72].

### Statistics

Data were compared using the student’s t-test. Comparisons between multiple groups were done using one- or two-way ANOVA with Tukey or Sidek’s post-test. Parasite load data determined by LDA and absolute number data were log transformed for graphical representation and statistical analysis. All p-values are two-tailed. Statistical calculations were done in Graphpad PRISM 9.0 (www.graphpad.com). * p < 0.05, ** p < 0.01, *** p < 0.005, **** p < 0.0001, n.s. = not significant.

### ELISA

Whole blood was collected using tail vein nicks following anti-IFN-γ (XMG1.2) treatment at 12, 28, 96, and 192 hours post-treatment. Serum was isolated and the presence of Rat IgG1 was assessed using the IgG1 Rat Uncoated ELISA Kit with Plates (Thermo Fisher, Catalog # 88-50500-22)

### Confocal intravital imaging and image analysis

Image analysis was performed as described previously [34,69,74]. Briefly, anesthetized mice were imaged in the lateral recumbent position, allowing the ventral side of the ear pinna to rest beneath a coverslip. Images were acquired using a Leica SP8 resonant scanning microscope (Leica Microsystems), equipped with a tunable white light laser, resonance scanner and a 25X 0.95 NA water immersion objective. A combination of HyD and PMT internal detectors were used for detection at 558–583 nm (dsRed), 488–509 nm (eGFP), and 630nm–650nm (Deep Red). Data were analyzed using Leica image analysis software.

### CD11b^+^ phagocyte purification

CD11b^+^ phagocytes were isolated from skin tissue from processing using CD11b MicroBeads UltraPure, mouse magnetic bead separation (Miltenyi Biotec) according to product protocol.

### qRT-PCR

Left and right ear cell homogenates were pooled and RNA was purified using an RNeasy mini kit according to the manufacturer’s protocol (Qiagen). Reverse transcription was performed with the High Capacity cDNA Reverse Transcriptional Kit (Thermo Fisher). Real-time PCR was performed on a StepOne Real-Time PCR System. Results were analyzed by the comparative threshold cycle method using 2^-ΔΔCT^ to determine fold change. Genes were normalize to the 18S rRNA endogenous control and to non-infected mice as a sample control. The mammalian TaqMan probes use can be found in the Key Resources Table.

## Supporting information

S1 Fig Needle versus infected sand fly challenge elicits similar responses at the site of secondary challenge. Mice were naïve or infected with 10^4^ *L*.*m*. s.c. in the left hind footpad (LHFP) and allowed to go chronic for 16 weeks. IFN-y production following antigen re-stimulation of ear dermis-derived CD4^+^ T cells was then assessed at 72 hours post-challenge with either 10^3^ *L*.*m*. metacyclic promastigotes or exposure to the bites of 4 *L*.*m*.-infected sand flies. **(A)** Representative flow plots of IFN-y^+^ producing dermal T cells. **(B)** Analysis of the frequency of IFN-γ^+^ cells within the TcRβ^+^CD4^+^ dermal population. n = 4 ears per group. ** p = 0.0067, Two-way ANOVA with Sidek’s post-test.
(TIF)

S2 Fig *Leishmania*-specific CD4^+^ T_EFFs_ retain high levels of Ly6C, variable levels of KLRG1, and low levels of PD-1 over the course of an intradermal infection. **(A-G)** Naïve C57Bl/6 mice were infected intradermally in both ears with 10^5^ *L*.*m*.-RFP. Ears, ear dLNs, and spleens were isolated at the indicated time points p.i.. **(A)** Ear lesions over the course of infection. **(B)** Representative CD44 vs tetramer, Ly6C vs CD62L, and KLRG1 vs PD-1 staining of the spleen, dLNs, and ears at 9 weeks p.i. in the column-bound fraction. **(C)** Representative CD44 vs tetramer, Ly6C vs CD62L, and KLRG1 vs PD-1 staining of the spleen, dLNs, and ears at 9 weeks p.i. in the column-unbound fraction. **(D)** Absolute number of PEPCK-specific T cells in the ear, ear dLN, and spleen over the indicated time course. **(E)** %Ly6C^+^ from PEPCK-sp tetramer^+^CD90.2^+^CD4^+^CD44^+^ T cells **(F)** %KLRG1^+^PD-1^-^ from PEPCK-sp tetramer^+^CD90.2^+^CD4^+^CD44^+^ T cells **(G)** %KLRG1^-^PD-1^+^ from PEPCK-sp tetramer^+^CD90.2^+^CD4^+^CD44^+^ T cells. Data is pooled from two independent experiments. Ears (n = 8–16), ear dLNs (n = 4–9), and spleens (n = 4–9).
(TIF)

S3 Fig Representative phagocyte flow cytometry gating strategy. Ear derived cells were stained with the indicated antibodies for analysis by flow cytometry. Cells were gated based on SSC-A and FSC-A to isolate leukocytes, followed by FSCA and FSC-W to isolate single cells, then dead cells were excluded using a LIVE/DEAD dye. Cell populations were then gated as depicted.
(TIF)

S4 Fig In chronic mice pre-existing cells with the capacity to produce IFN-y upon antigen-exposure reside in unchallenged dermal sites. Mice were naïve or infected with 10^4^ *L*.*m*. s.c. in the left hind footpad (LHFP) and allowed to go chronic for 10–16 weeks. Cytokine production following antigen-restimulation of ear dermis-derived CD4+ T cells was then assessed. **(A)** Representative flow plots of IFN-y^+^TNF-α^+^ producing T cells after re-stimulation of dermal cells prior to (right ears), or following (left ears) perfusion as outlined in the schematic. **(B)** Quantitative analysis of the number of IFN-y cells in perfused and non-perfused dermal, non-challenged sites from chronic versus naive mice. In the skin, perfusion efficiently removes circulating cells based on i.v. labelling [20].
(TIF)

S5 Fig The number of *L*. *major* infected cells does not increase over the first 4 days of infection. Mice were infected with 2 x 10^5^ *L*.*m*.-RFP i.d. in both ears and assessed at the indicated time points. **(A)** # of specified populations per ear over the indicated time course. n = 6–8 ears/time point.
(TIF)

S6 Fig iNOS production in inflammatory monocytes populations after D0 and D-4 adoptive transfer of Th1 cells. Frequencies of the specified cell populations over the course of the kinetic described in Fig 5. Data are pooled from 2 independent experiments, n = 6–9. Error bars are +/- SD. (*) p < 0.05, (**) p < 0.01, (***) p < 0.005, (****) p < 0.0001, n.s. = not significant.
(TIF)
