# Supplementary figures and images for: Protective CD4+ Th1 cell-mediated immunity is reliant upon execution of effector function prior to the establishment of the pathogen niche
Source: PLoS Pathog. 2021 Sep 20;17(9):e1009944. doi: 10.1371/journal.ppat.1009944 (PMC8483310; doi:10.1371/journal.ppat.1009944)

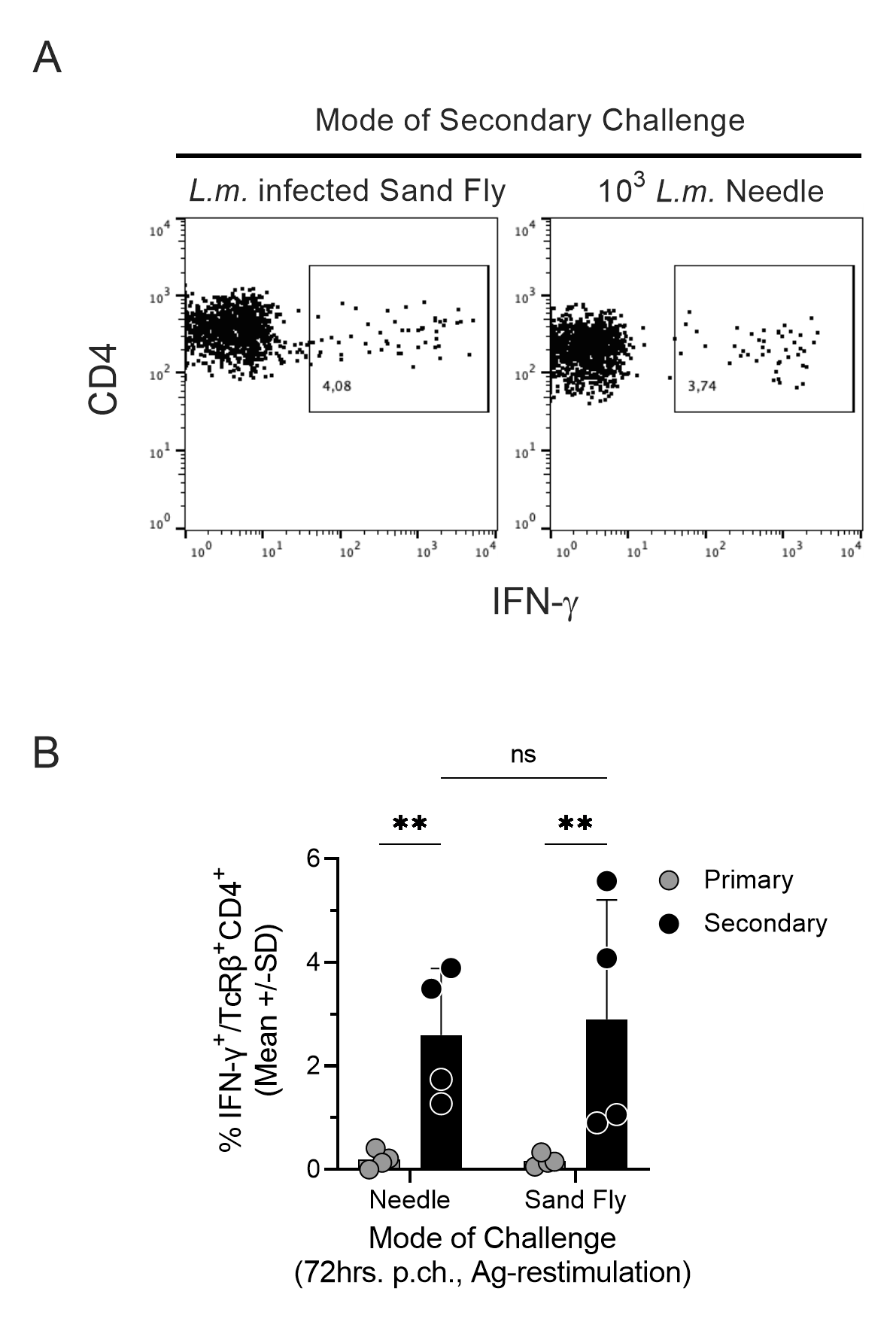

Supplement: S1 Fig — Mice were naïve or infected with 104 L.m. s.c. in the left hind footpad (LHFP) and allowed to go chronic for 16 weeks. IFN-y production following antigen re-stimulation of ear dermis-derived CD4+ T cells was then assessed at 72 hours post-challenge with either 103 L.m. metacyclic promastigotes or exposure to the bites of 4 L.m.-infected sand flies. (A) Representative flow plots of IFN-y+ producing dermal T cells. (B) Analysis of the frequency of IFN-γ+ cells within the TcRβ+CD4+ dermal population. n = 4 ears per group. ** p = 0.0067, Two-way ANOVA with Sidek’s post-test. (TIF) [file ppat.1009944.s001.tif]

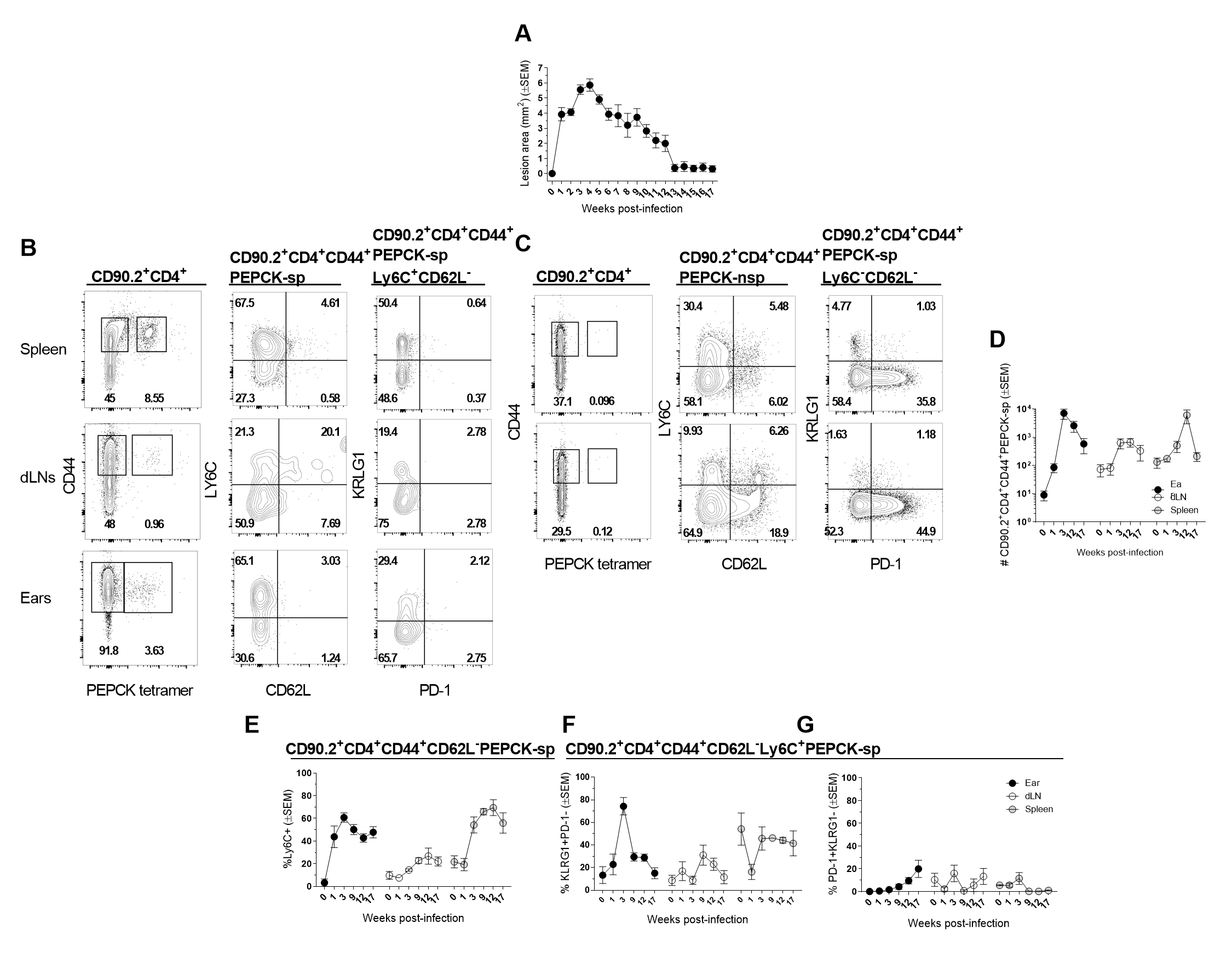

Supplement: S2 Fig — (A-G) Naïve C57Bl/6 mice were infected intradermally in both ears with 105 L.m.-RFP. Ears, ear dLNs, and spleens were isolated at the indicated time points p.i.. (A) Ear lesions over the course of infection. (B) Representative CD44 vs tetramer, Ly6C vs CD62L, and KLRG1 vs PD-1 staining of the spleen, dLNs, and ears at 9 weeks p.i. in the column-bound fraction. (C) Representative CD44 vs tetramer, Ly6C vs CD62L, and KLRG1 vs PD-1 staining of the spleen, dLNs, and ears at 9 weeks p.i. in the column-unbound fraction. (D) Absolute number of PEPCK-specific T cells in the ear, ear dLN, and spleen over the indicated time course. (E) %Ly6C+ from PEPCK-sp tetramer+CD90.2+CD4+CD44+ T cells (F) %KLRG1+PD-1- from PEPCK-sp tetramer+CD90.2+CD4+CD44+ T cells (G) %KLRG1-PD-1+ from PEPCK-sp tetramer+CD90.2+CD4+CD44+ T cells. Data is pooled from two independent experiments. Ears (n = 8–16), ear dLNs (n = 4–9), and spleens (n = 4–9). (TIF) [file ppat.1009944.s002.tif]

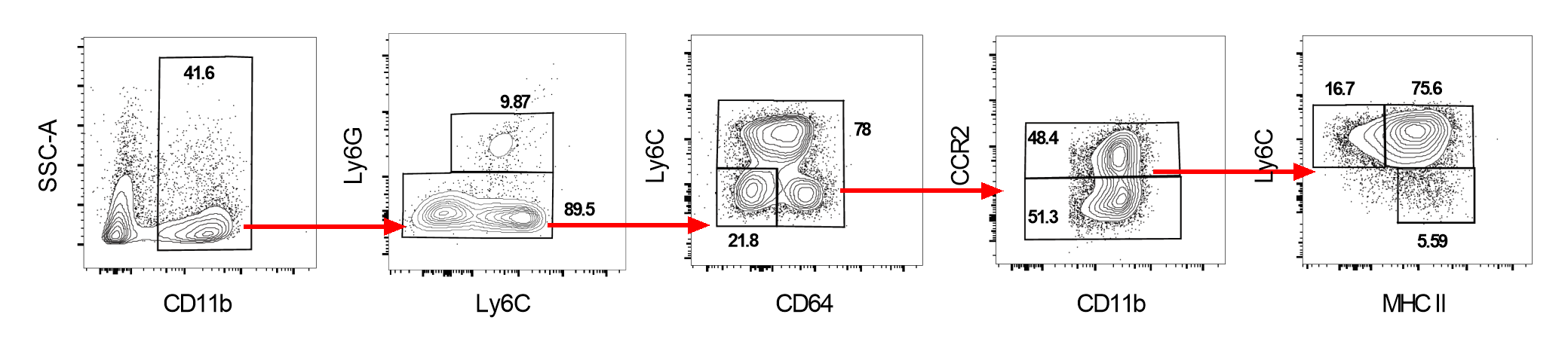

Supplement: S3 Fig — Ear derived cells were stained with the indicated antibodies for analysis by flow cytometry. Cells were gated based on SSC-A and FSC-A to isolate leukocytes, followed by FSCA and FSC-W to isolate single cells, then dead cells were excluded using a LIVE/DEAD dye. Cell populations were then gated as depicted. (TIF) [file ppat.1009944.s003.tif]

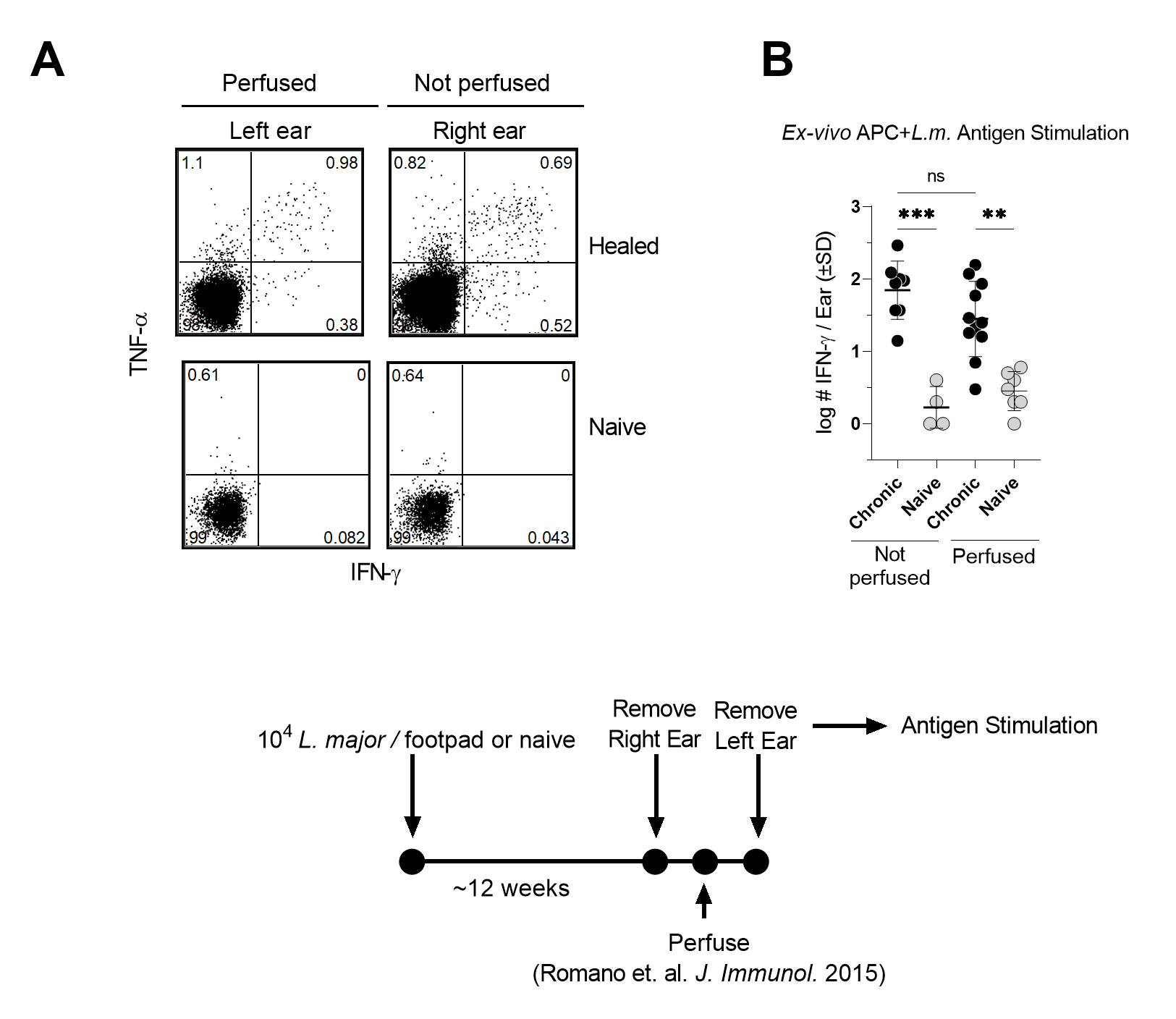

Supplement: S4 Fig — Mice were naïve or infected with 104 L.m. s.c. in the left hind footpad (LHFP) and allowed to go chronic for 10–16 weeks. Cytokine production following antigen-restimulation of ear dermis-derived CD4+ T cells was then assessed. (A) Representative flow plots of IFN-y+TNF-α+ producing T cells after re-stimulation of dermal cells prior to (right ears), or following (left ears) perfusion as outlined in the schematic. (B) Quantitative analysis of the number of IFN-y cells in perfused and non-perfused dermal, non-challenged sites from chronic versus naive mice. In the skin, perfusion efficiently removes circulating cells based on i.v. labelling [20]. (TIF) [file ppat.1009944.s004.tif]

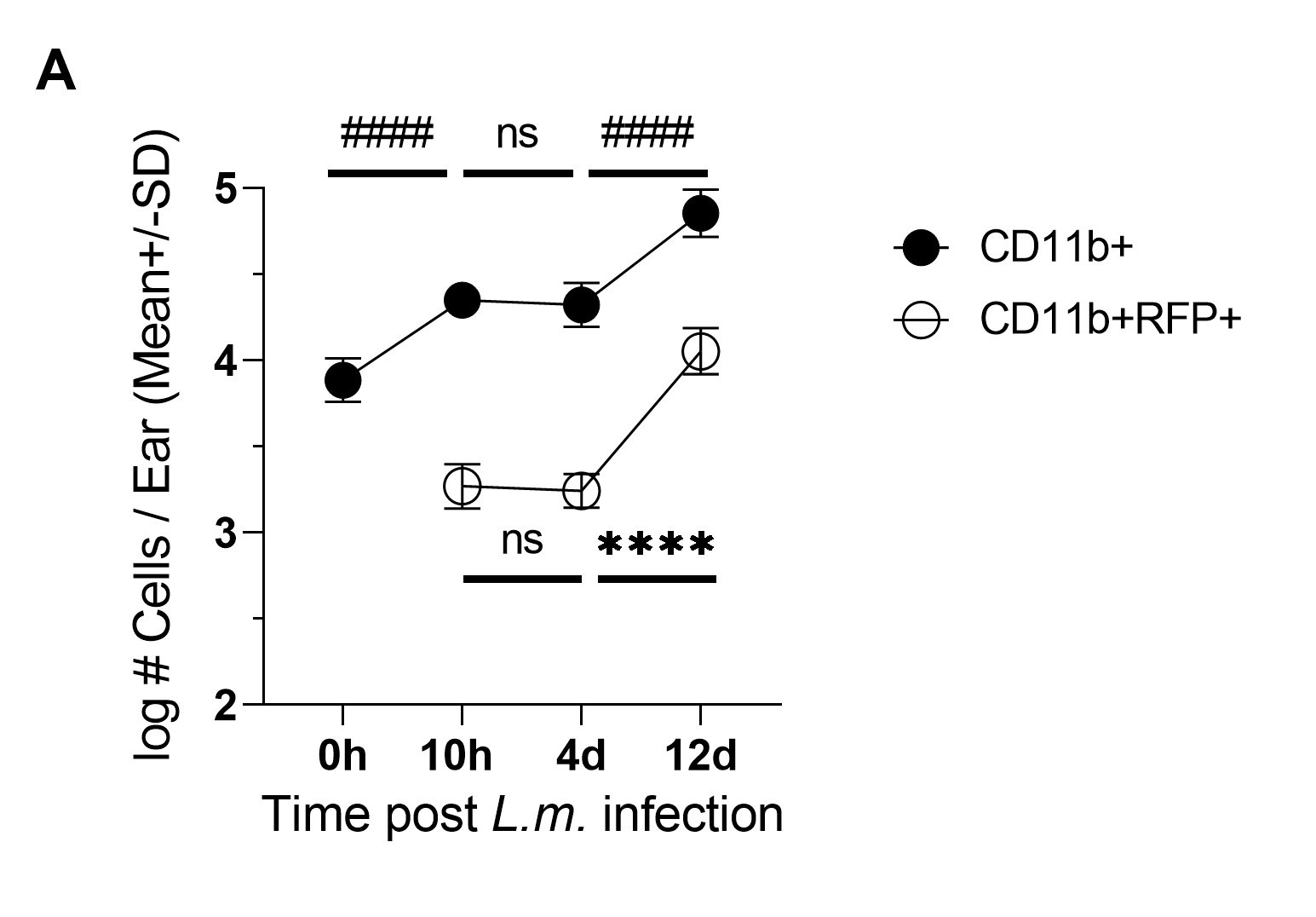

Supplement: S5 Fig — Mice were infected with 2 x 10^5^ L.m.-RFP i.d. in both ears and assessed at the indicated time points. (A) # of specified populations per ear over the indicated time course. n = 6–8 ears/time point. (TIF) [file ppat.1009944.s005.tif]

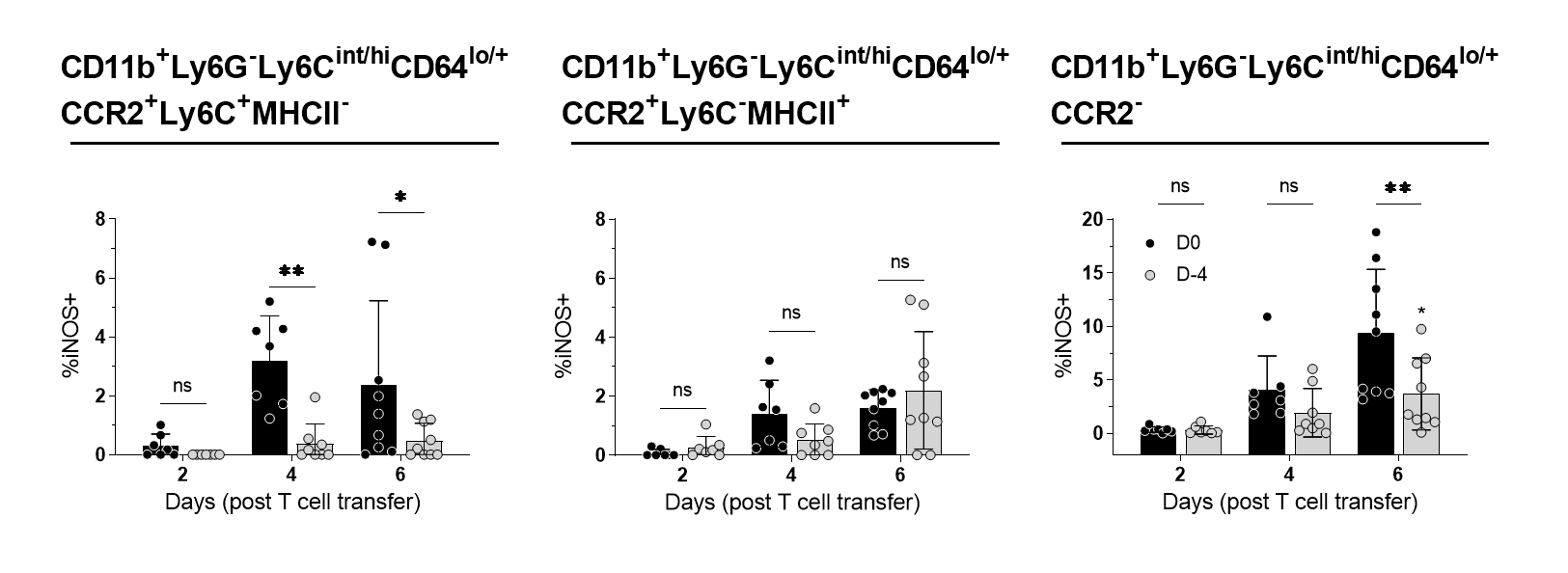

Supplement: S6 Fig — Frequencies of the specified cell populations over the course of the kinetic described in Fig 5. Data are pooled from 2 independent experiments, n = 6–9. Error bars are +/- SD. (*) p < 0.05, (**) p < 0.01, (***) p < 0.005, (****) p < 0.0001, n.s. = not significant. (TIF) [file ppat.1009944.s006.tif]
